# Strain dynamics of contaminating bacteria modulate the yield of ethanol biorefineries

**DOI:** 10.1038/s41467-024-49683-2

**Published:** 2024-06-22

**Authors:** Felipe Senne de Oliveira Lino, Shilpa Garg, Simone S. Li, Maria-Anna Misiakou, Kang Kang, Bruno Labate Vale da Costa, Tobias Svend-Aage Beyer-Pedersen, Thamiris Guerra Giacon, Thiago Olitta Basso, Gianni Panagiotou, Morten Otto Alexander Sommer

**Affiliations:** 1 The Novo Nordisk Foundation Center for Biosustainability, Technical University of Denmark, Kongens Lyngby, 2800 Denmark; 2 School of Chemistry and Molecular Biosciences, University of Queensland, St Lucia, 4072 Australia; 3 Leibniz Institute for Natural Product Research and Infection Biology, Jena, 07745 Germany; 4 Escola de Engenharia de Alimentos da Universidade de Campinas, 13083-862 Campinas, SP Brazil; 5 Departamento de Engenharia Química da Escola Politécnica da Universidade de São Paulo. Universidade de São Paulo, 05508-000 São Paulo, SP Brazil

**Keywords:** Applied microbiology, Metabolic engineering, Microbial ecology, Microbiome

## Abstract

Bioethanol is a sustainable energy alternative and can contribute to global greenhouse-gas emission reductions by over 60%. Its industrial production faces various bottlenecks, including sub-optimal efficiency resulting from bacteria. Broad-spectrum removal of these contaminants results in negligible gains, suggesting that the process is shaped by ecological interactions within the microbial community. Here, we survey the microbiome across all process steps at two biorefineries, over three timepoints in a production season. Leveraging shotgun metagenomics and cultivation-based approaches, we identify beneficial bacteria and find improved outcome when yeast-to-bacteria ratios increase during fermentation. We provide a microbial gene catalogue which reveals bacteria-specific pathways associated with performance. We also show that *Limosilactobacillus fermentum* overgrowth lowers production, with one strain reducing yield by ~5% in laboratory fermentations, potentially due to its metabolite profile. Temperature is found to be a major driver for strain-level dynamics. Improved microbial management strategies could unlock environmental and economic gains in this US $ 60 billion industry enabling its wider adoption.

## Abstract (web summary)

Industrial bioethanol production is driven by ecological interactions within the microbial community rather than the interactions within yeast population. Here, the authors identify bacteria affecting ethanol production and reveal temperature as the major driving force for strain-level dynamics.

## Introduction

To date, contaminant microbes have been characterised at limited genomic resolution using culture-based methods that did not capture true microbial diversity, as well as meta-barcoding of microbial communities, albeit only at selected process steps and providing limited insight into the consequences of observed changes in composition^1–6^.

The true impact of non-yeast microbes on industrial fermentation performance is unclear – that is, whether it is effected by strains, species or at community level, as well as the underlying functions and ecological factors that enable this. Indeed, laboratory studies of lactic acid bacteria indicate their effects on yeast are species-specific, and some could in fact be beneficial (e.g. *Lactobacillus amylovorus*)^7–9^. However, accurate assessment of bioconversion performance in an industrial context is complicated by the fluctuating nature of the system^10^. It is accepted that current methods centred on ethanol yield are oversimplified and should be considered in the context of, for example, viability of the yeast cells and other resident microbiota in the fermentations^11^.

Biofuels have assisted in the transition from fossil fuels to an electrified transportation grid in developing economies. Use of bioethanol as an alternative source of energy supply is a major focus of global strategies to reduce greenhouse gas (GHG) emissions. In Brazil, considered to have the most successful biofuels programme in the world, its use in place of gasoline is estimated to have lowered GHG emissions by >60%^12,13^.

Over 80% of renewable fuels worldwide comes from bioethanol derived from yeast fermentation, which entails the conversion of sugars in plant feedstock (such as sugarcane or corn) to ethanol^14^. However, yield levels have been hampered by factors intrinsic to the bioprocess at industrial scale. Continual changes in oxygen levels, temperature, pH, sugar and ethanol concentration, for example, create a non-optimal environment for yeast to thrive^15^.

Industrial bioethanol production is driven by the yeast *Saccharomyces cerevisiae*, in the presence of other microorganisms from the raw biomass. Mainly comprised of bacteria, these microbes are often regarded as contaminants that compete for resources and lower fermentation efficiency^16^, causing an estimated 3% reduction in ethanol yield (equating to over 960 million litres per year in Brazil alone)^1^. Biorefineries often mitigate this by including an acid-wash step to remove bacteria from the yeast biomass before re-introducing it to the fermentation vessels^15^. Other approaches include the use of broad-spectrum antibiotics and other antimicrobial compounds. However, these approaches do not fully address the issue and an effective strategy that is economically and environmentally sustainable remains elusive^7,15–17^.

In this study, we describe and compare the microbial populations of the industrial bioethanol process across all unitary steps at two major biorefineries in Brazil. Sampled at 3 time points over a single production season, we use a combination of shotgun metagenomics and cultivation-based methods to interrogate the microbiome at multiple taxonomic and functional levels and identify ecological factors underpinning community dynamics and bioconversion efficiency. We link increased temperatures with growth of specific bacteria that impede yeast viability and fermentation performance. Furthermore, strains from the same bacterial species showed distinct effects, most likely driven by metabolic differences, that can be beneficial or detrimental to ethanol yield. Our findings motivate the adoption of higher resolution methods to holistically monitor the microbial communities in industrial-scale fermentation processes in order to maintain performance. We also suggest strategies towards the development of strategies to control the growth of undesirable microbes, which will make bioethanol production more cost-effective and thus encourage greater adoption of this renewable energy source.

## Results

### A comprehensive resource to study microbiome impact on bioethanol production yields

**Fig. 1:**
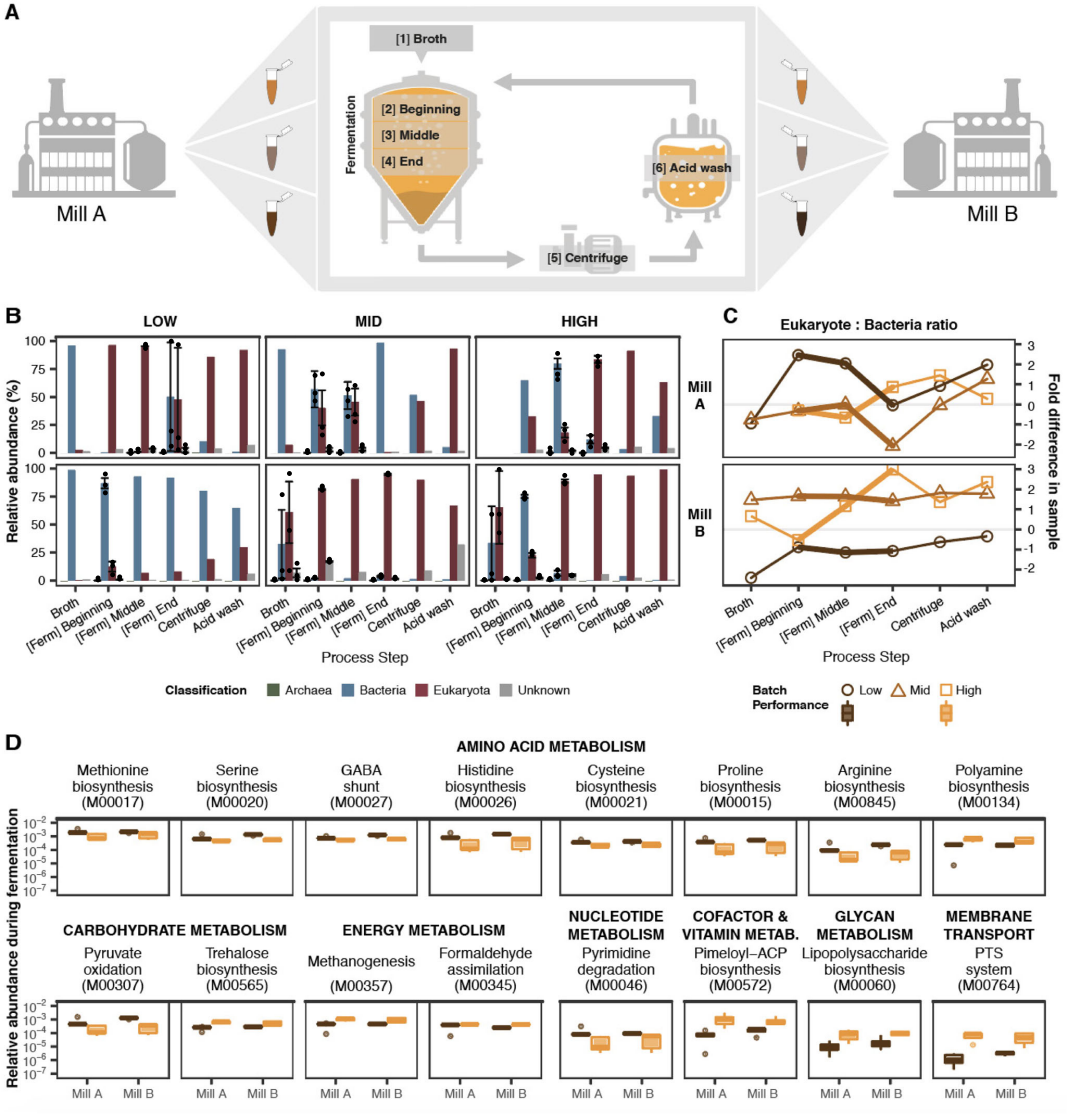
Microbial dynamics during fermentation influence the performance of industrial bioethanol production. **A** Outline of study sampling strategy, repeated 3 times in a single production season at 2 independent mills. Fresh media [1] are fed into fermentation vessels for 3 h. Samples were collected at 0–1.5 h [2], 1.5–3 h [3] and post-feeding [4]. Biomass is then centrifuged [5] and acid-washed [6] before re-entering the vessels. Vector images were obtained from Flaticon (www.flaticon.com), and figure as created using Adobe Illustrator. **B** Metagenomic profiling of microbial communities, grouped by relative batch performance (columns) per mill (row). Higher numbers of bacteria (blue) or eukaryotes (red) are not linked to better production performance. One sample from Mill A (starting broth, high-performing batch) did not contain sufficient DNA for sequencing. Error bars show variation across multiple samples, where applicable. For samples containing more than one datapoint, *n* = 3 biologically independent samples. Data are presented as mean values +/- SD. **C** Eukaryote-to-bacteria ratios across the production process. Each batch is connected by a line. In both mills, eukaryotic populations increased in the high-performing batches (orange, square) during fermentation steps (thick line) and decreased in lower-performing batches (light and dark brown, circle and triangle). Grey line denotes equal proportion of eukaryotes and bacteria (i.e. zero-fold difference). The *y*-axis indicates the fold change in the eukaryote to bacteria ratio. Higher fold values indicate a greater eukaryote abundance, and lower fold values a higher prokaryote abundance. **D** Genes associated with changes in fermentation performance. In both mills, the relative abundance of these 16 sets, involved in metabolism or membrane transport, differed between low- (brown) and high-performing (orange) batches (FDR < 0.1 for both mills). Increase in genes linked to bacteria-specific pathways, e.g. lipopolysaccharide biosynthesis and the phosphotransferase (PTS) system, is associated with better performance. Gene modules are ordered by decreasing relative abundance. KEGG Module IDs are provided in parentheses. For samples containing more than one datapoint, *n* = 3 biologically independent samples. The centre line denotes the median value (50th percentile), while the box contains the 25th–75th percentiles of dataset. The whiskers mark the 5th and 95th percentiles, and values beyond these upper and lower bounds are considered outliers, marked with dots. Source data are provided as a Source Data file.

A total of 56 samples were collected from two independently operated sugarcane ethanol biorefineries in Brazil, hereafter referred to as Mills A and B (Fig. 1A, Methods, Supplementary Data 1). The mills were selected as they have similar production capacity, are situated in regions of comparable climate, and use sugarcane harvested from different areas. Both biorefineries deploy the Melle-Boinot fermentation process, whereby ethanol is produced via fast, high cell-density, fed-batch fermentations in a series of unidirectional steps^15^—providing defined sampling points for our study. Notably, yeast biomass is recovered after each cycle and reused (following dilution with water and an acid-wash step) for as many as 750 fermentation batches per year^18^. To account for potential differences caused by seasonal variation, we sampled each mill at three distinct timepoints over a single production season (Supplementary Data 1).

Shotgun metagenomic sequencing was applied to the 56 samples, providing >2.8 × 10^5^ Gbp of high-quality data (Methods, Supplementary Data 2). These were assembled into contiguous sequences, from which we created a non-redundant catalogue of 296,257 genes derived from the bioethanol production process microbiome (Methods). Gene lengths ranged from 18–5,822aa (mean 162.8 ± 165.3) (Supplementary Fig. 1**)**.

Measurements commonly used by the industry to assess bioethanol production process quality were collected at each sampling to describe each fermentation batch (Supplementary Data 1). Important to mention that, due to confidentiality reasons, not all process data has been provided, leading to possible confounders without proper data available. To facilitate evaluation and fair comparison of performance across the different batches, we devised a composite metric that accounted for ethanol yield, quality of biological catalysts as well as potential inhibitors of fermentation performance (Methods). This enabled the three batches from each biorefinery to be ranked by performance: low, mid and high.

### Dynamics between yeast and bacteria drive industrial fermentation performance

To capture the diversity of the microbial communities involved in bioethanol production, we used a bioinformatic approach to detect known and putative eukaryotic, bacterial and archaeal species, utilising small and large subunit rRNA genes detected across our metagenomic samples (Fig. 1B, Methods). This method of taxonomic profiling revealed the microbiome to be primarily comprised of eukaryotic and bacterial populations in fluctuating quantities across the industrial process (Mill A: 52.7 ± 36.3% and 44.2 ± 37.5%; Mill B: 58.3 ± 39.6% and 36.4 ± 42.4%, respectively; Supplementary Data 1 and 2). The composition of the starting broth (mixture of sugarcane molasses and concentrated juice) showed highest intra- and inter-variability across our samples. *Saccharomyces* species were most prevalent and abundant across the production process (Mill A: 52.6 ± 36.3%, Mill B: 58.1 ± 39.9%), and we observed that acid-wash steps resulted in reduction (but not complete removal) of bacteria (Fig. 1B, Supplementary Data 1 and 2). Surprisingly, high-performing batches were not characterised by a dominance in eukaryotes; low-performing batches were not necessarily dominated by bacteria. In both biorefineries, we observed that better performance was linked to an increasing eukaryote-to-bacteria ratio during fermentation, irrespective of which of the two microorganisms was initially more abundant (Fig. 1C).

Functional profiling of the microbial communities, using the constructed gene catalogue, revealed 16 sets of genes (KEGG Modules; functional units within a pathway) that are linked to changes in fermentation performance, observed consistently in both biorefineries (FDR < 0.1 in each mill) (Fig. 1D, Methods). Many were components of pathways highly conserved in microbes, for example, amino acid biosynthesis and carbohydrate metabolism. Interestingly, genes involved in lipopolysaccharide (LPS) biosynthesis and the phosphotransferase (PTS) system, which are unique to bacteria and not found in eukaryotes, were associated with better performance. However, increases in PTS and LPS were not directly linked to corresponding increases in bacteria (Fig. 1B), suggesting that changes were also occurring within the bacterial community. Together, these observations point to a central role of microbial community dynamics in driving the success of the bioconversion process.

### Lactic acid bacteria dominate the contaminant microbial community and their interplay influences process performance

**Fig. 2:**
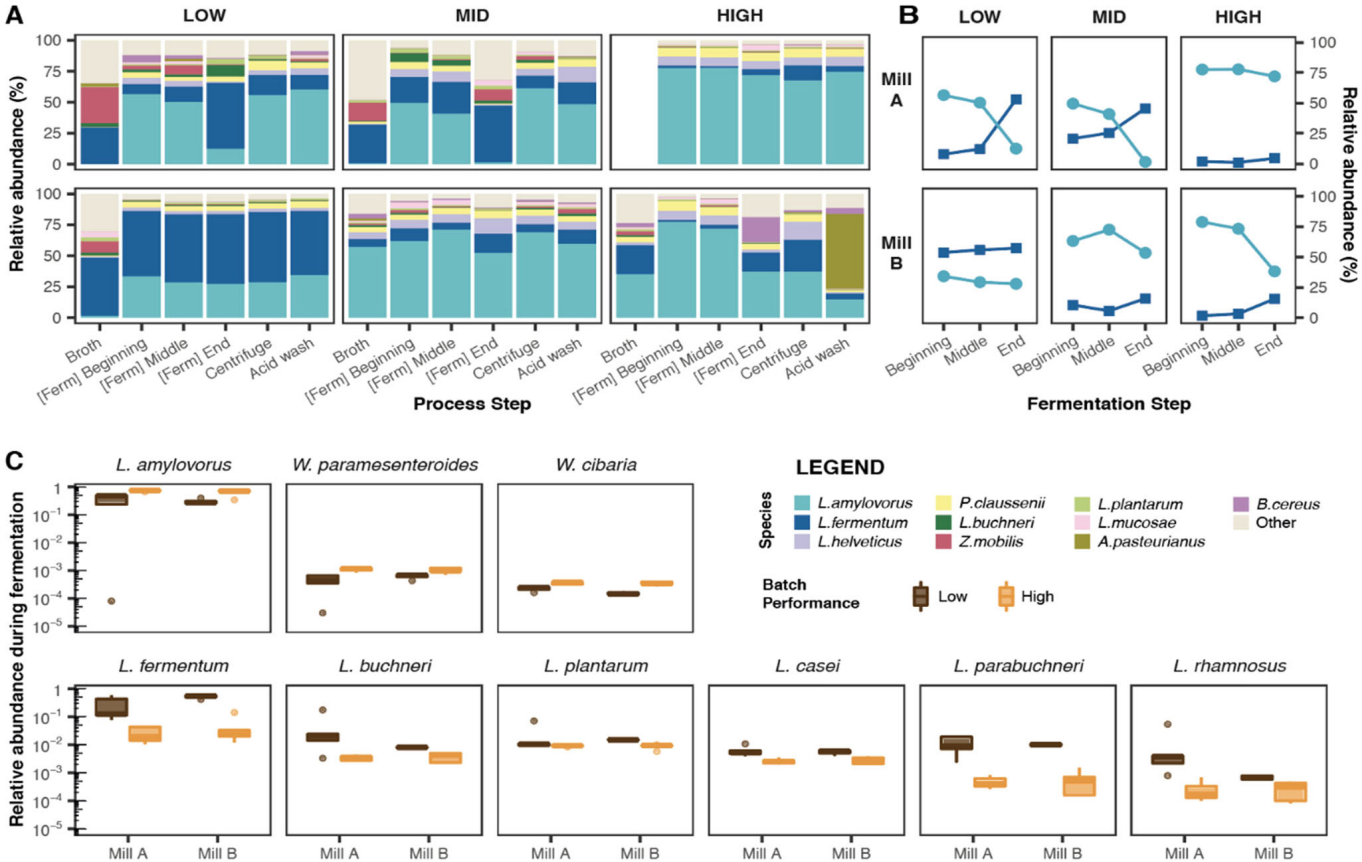
*L. amylovorus* and *L. fermentum* are the dominant bacteria in the industrial bioethanol microbiome and their interplay is linked to production performance. **A** Taxonomic profiling of bacterial populations detected across process steps, grouped by relative batch performance (columns) per mill (row). *L. amylovorus* (light blue) and *L. fermentum* (dark blue) strains comprise the majority of bacteria across samples. Bacterial communities in the high-performing batch of Mill A and low-performing batch of Mill B undergo less change compared to others. For visual clarity, the 10 most abundant species are shown; beige colour represents other bacteria. Average values were used in process steps where multiple samples were collected. **B** Relative abundance of *L. amylovorus* and *L. fermentum* during fermentation steps. In both mills, high-performing batches contained more *L. amylovorus* strains. Across all batches and mills, *L. fermentum* increases and *L. amylovorus* decreases by the end of fermentation. **C** Bacterial species associated with changes in fermentation performance. High-performing batches showed increases in *L. amylovorus* and *Weisella* species (top row), while other lactic acid bacteria including *L. fermentum*, *L.buchneri* and *L.plantarum* decreased (bottom row) (FDR < 0.1 in both mills). Species are ordered by decreasing relative abundance. For samples containing more than one datapoint, *n* = 3 biologically independent samples. The centre line denotes the median value (50th percentile), while the box contains the 25th to 75th percentiles of dataset. The whiskers mark the 5th and 95th percentiles, and values beyond these upper and lower bounds are considered outliers, marked with dots. Source data are provided as a Source Data file.

To elucidate the role of bacterial populations on performance, we profiled our samples using a higher resolution genome-based approach (Fig. 2A, Methods). A total of 48 bacterial species, covering 5 phyla, were identified in our samples (Supplementary Table 1). Comparing across all process steps and timepoints, we found that stability of the bacterial community was not linked to batch performance, and community composition also did not significantly differ between the two biorefineries (*p* = 0.29, PERMANOVA). Bacteria in the starting broth were again the most diverse and distinct to other steps in the bioethanol production process (Supplementary Fig. 2).

Lactobacillaceae species were the most abundant bacteria, as supported by literature^1,4,7,19,20^, in all but one sample (Fig. 2A). *Lactobacillus amylovorus* and *Limosilactobacillus fermentum* constituted >50% of bacteria in these samples and are described as key contaminants in a number of ethanol fermentation processes^1,21–23^. However, in both mills we found higher production performance to be associated with increases in *L. amylovorus* and *Weissella* species (*W. paramesenteroides*, *W. cibaria*), and decreases in *L. fermentum*, *Lentilactobacillus buchneri*, *Lactiplantibacillus plantarum* and other lactic acid bacteria (Wilcoxon rank-sum test, FDR < 0.1; Fig. 2B-C). Interestingly, we also observed an inverse relationship between the two dominant species during the fermentation steps, whereby decreases in *L. amylovorus* coupled with an increase in *L. fermentum* (Spearman’s *ρ* = −0.91, FDR = 4 × 10^−14^; Fig. 2B). This was especially evident in lower-performing batches and suggests ecological interplay between the two bacteria (partially motivated by differences in physiology^1^ and ethanol tolerance^4^) may play a role in driving the efficiency of bioethanol production. Indeed, *L. amylovorus* was previously described to interact with *S. cerevisiae* via cross-feeding, benefiting yeast metabolism and in turn increasing ethanol yield^8^.

### Increases in *L. fermentum* and other bacteria are linked to fermentation conditions detrimental to yeast

**Fig. 3:**
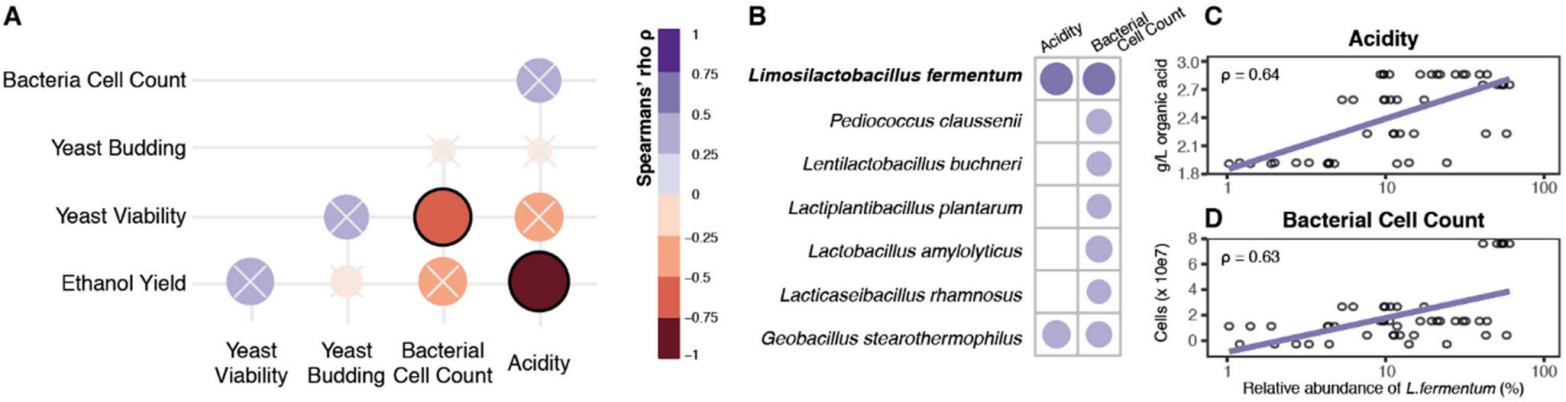
*L. fermentum* and other bacteria are associated with indicators of low fermentation performance. **A** Correlation matrix of industrial fermentation parameters that showed strong associations throughout the production season (all sampling timepoints). Size and colour of points show correlation strength and direction, respectively. Low ethanol yield is associated with high acidity titres (dark red, Spearman’s *ρ* = −0.84), and increased bacterial cell count is linked to lower viability of yeast cells (orange, Spearman’s *ρ* = −0.72). Cross denotes correlations with FDR > 0.1. **B** Bacterial species associated with fermentation performance parameters. Species are ordered by decreasing relative abundance. **C** Increased *L. fermentum* is linked to higher acidity titres (Spearman’s *ρ* = 0.64, FDR < 0.05), and increased bacterial cell count (**D**; Spearman’s *ρ* = 0.63, FDR < 0.05). Source data are provided as a Source Data file.

To identify potential drivers of high performance, pairwise correlation analysis was conducted on the quality indicators collected for each sample (Fig. 3A, Methods). We found that batches with lower acidity titres were associated with higher ethanol yields (Spearman’s *ρ* = −0.84, FDR = 2 × 10^−5^), consistent with laboratory-scale findings^24^. Similarly, batches with more bacteria tended to have less viable yeast cells (Spearman’s *ρ* = −0.72, FDR = 2 × 10^−3^). These results are in line with previous findings^6^.

Incorporating these findings with our microbiome data revealed potential links between observed changes in the microbial communities and batch performance (Fig. 3B, Methods). Of the 48 bacterial species detected across our samples, seven (all Gram-positive Firmicutes) were linked to increased acidity and/or bacterial cell count during fermentation, with *L. fermentum* correlating most strongly (acidity *ρ* = 0.64, FDR = 9 × 10^−5^; bacterial cell count ρ = 0.63, FDR = 2 × 10^−4^; Fig. 3C, D). This is supported by a recent study that demonstrated the detrimental effects of acetic acid produced by *L. fermentum* on yeast in corn-based fermentations^9^.

Our analysis also revealed bacteria with no known links to industrial bioethanol production performance, such as *Geobacillus stearothermophilus*. This species is a thermophile capable of producing a vast array of cellulolytic enzymes^25^. Its presence in the fermentation process also suggests that this environment may be an untapped source for novel industrially-relevant enzymes.

### Metabolic differences drive strain-specific impact on fermentation performance

**Fig. 4:**
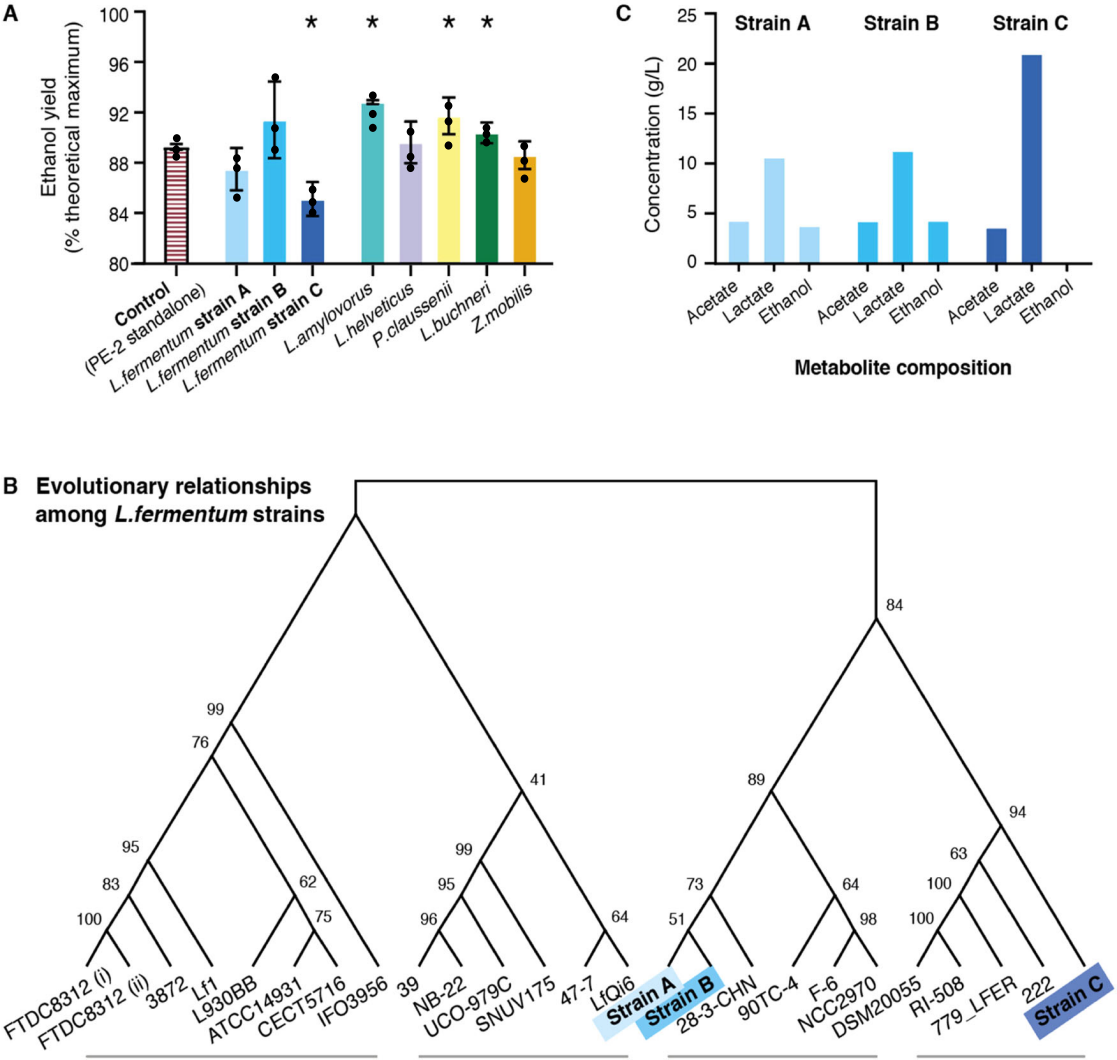
Specific *L. fermentum* strains reduce bioethanol production yield, possibly due to metabolic differences. **A** Ethanol yield from pairwise fermentations of industrial yeast strain PE-2 with 3 *L. fermentum* industrial isolates (blue) and the 5 most abundant bacteria in the bioethanol production microbiome, compared to standalone fermentation (white). Ethanol yields were enhanced by *L. amylovorus*, *P. claussenii* and *L. buchneri*, and reduced only by *L. fermentum* strain (**C**) (*p < 0.05). For all experiments, *n* = 3 biologically independent samples. Data are presented as mean values +/- SD. Final ethanol yields were compared by multiple *t-*test (statistical significance analysis with alpha value of 0.05). **B** Cladogram of the 3 *L. fermentum* isolates and other published *L. fermentum* genomes, based on the alignment of 40 bacterial marker genes. *L. fermentum* strains broadly cluster into 4 groups; strain (**C**) belongs to a different clade than strains (**A**, **B**). Internal nodes are labelled with bootstrap values from 500 resamplings. Image generated using MEGAX, with the triangular root replaced for visual clarity. **C** Metabolite profiles measured from the supernatant of the 3 *L. fermentum* industrial isolates. Strains A and B showed similar production of acetate, lactate and ethanol. Strain C (dark blue, right) produced no ethanol and almost twice as much lactate as strains A and B. Source data are provided as a Source Data file.

Given the link observed between *L*. *fermentum* and poor fermentation performance, we sought to establish if this phenomenon applied to all, or a subset of, *L*. *fermentum* strains. To elucidate the impact of strain variation on ethanol yield, we performed static batch cultivations using the industrial *S. cerevisiae* strain PE-2 and three unique *L. fermentum* strains isolated directly from our samples, hereafter referred to as strains A, B and C (Fig. 4A, Methods). Here, we conducted pairwise fermentations that simulated the conditions of a typical industrial setup, using a chemically semi-defined medium that resembled sugarcane molasses-based broth^2,26^ and yeast-to-bacteria ratio of 100:1^16^. To contextualise our findings, we also included the five most abundant bacteria identified in this study. Collectively, these six species accounted for >80% of known bacteria across our samples (Supplementary Data 3).

Our results indicated that different bacterial species can have varied effects on bioethanol production (Fig. 4A). The addition of *L. amylovorus*, *L. buchneri* or *Pediococcus claussenii* resulted in increased ethanol yield, in contrast with negligible impact observed for *Lactobacillus helveticus* and *Zymomonas mobilis*, when compared to standalone fermentation by yeast strain PE-2 (multiple *t*-test, *p* < 0.05). Both *L. amylovorus* and *P. claussenii* have a homofermentative metabolism, which has been shown to be less detrimental to *S. cerevisiae* in this fermentation setup^1^. Notably, *L. buchneri* strains have demonstrated heterofermentative properties, producing considerable amounts of ethanol and lactate from glucose^27^. Although this additional means of ethanol production can contribute to greater ethanol titres and yields, overgrowth of *L. buchneri* is linked to increased bacterial cell count and, in turn, decreased yeast viability, as observed in our metagenomics analyses (Figs. 2B, 3A, B).

Strikingly, our *L. fermentum* isolates also displayed varying effects on fermentation yield, with one strain reducing ethanol yield by 4.63 ± 1.35% (Fig. 4A; multiple *t*-test, *p* < 0.05). Phylogenomic analysis based on taxonomic bacterial marker genes (alongside published *L. fermentum* genomes) suggests that although all three strains were isolated from the same setting, this detrimental strain C belongs to a separate clade to strains A and B (Fig. 4B, Supplementary Fig. 3, Supplementary Data 3, Methods).

Metabolite profiling and co-cultivation experiments revealed potential mechanisms underlying the distinct traits observed in the *L. fermentum* isolates (Fig. 4C, Supplementary Table 1, Methods). We found that strain C had a markedly different metabolite profile: it did not produce ethanol and made twice as much lactate (as much as 21 g/L) with no corresponding change in acetate, thereby changing the overall ratio between these organic acids. This, as well as higher organic acid titres, was previously described to inhibit bioconversion performance of *S. cerevisiae*^9,21,28^. Moreover, the isolates also demonstrated strain-dependent effects on yeast growth. Co-cultivations between yeast and strain C resulted in a reduction of >23% of the yeast population, when compared to standalone controls (Supplementary Table 2). By contrast, strains A and B reduced yeast cells by only 7.4% and 6.5%, respectively. Taken together, our findings suggest that the inhibition of yeast ethanol production by lactic acid bacteria is mediated not by entire species but at strain-level, facilitated by differences in organic acid production profile^1^ and mechanisms that allow them to adapt more readily to a changing environment and influence yeast growth and metabolism.

After observing the significant differences in ethanol yield between the three *L. fermentum* strains during pairwise cultivations, we sought to further explore how the metabolic profile of both these isolates and the yeast strain might impact their physiology. We compared the growth profiles (final OD and specific growth rate; *μ*) of the yeast *S. cerevisiae* PE-2 inoculated in (1) diluted sugarcane molasses (20 g/L TRS) against molasses previously fermented with each of the 3 *L. fermentum* strains (2) with or (3) without the supplementation of sugars (to restore the original sugar titres) and, (4) with fresh molasses spiked with key bacterial metabolites (i.e. acetic and lactic acids). As expected, the cultivation in fresh molasses achieved the highest OD and μ values, and the lowest values were observed in the depleted molasses from microbial cultivation. However, there was a significant difference between the previously lactobacilli fermented molasses supplemented with a mixture of sucrose, glucose and fructose, and the fresh molasses spiked with bacterial metabolites, where higher ODs were achieved in the latter (Supplementary Fig. 4). Based on these observations, it seems that the inhibition of yeast growth is not solely driven by organic acid production by the bacteria, but probably by the lack of essential nutrients in the media that was previously cultivated with bacteria, suggesting competition for nutrients among them.

Final ODs (Supplementary Fig. 5) and specific growth rates (Supplementary Fig. 6) presented divergent absolute values between the different spent media produced by the 3 bacterial strains, with strain C resulting in lower growth rate, but higher OD values in fermented molasses supplemented with sugars, when compared to the neutral strain B. This raises the question whether the inhibitory effect of such strain is not somewhat related with the production of another inhibitory molecule, instead of simple nutrient depletion.

Considering the bidirectionality of microbial interactions, we decided to further evaluate the impact of yeast metabolism on the growth of the *L. fermentum* isolates. For that, the cultivation of the isolates in (1) freshly diluted molasses (20 g/L TRS) was compared against (2) depleted molasses from yeast cultivation; (3) depleted molasses from yeast cultivation supplemented with a mixture of sucrose, glucose and fructose (to restore the original sugar titres); and (4) molasses spiked with yeast metabolites (i.e. ethanol and glycerol). Interestingly, the results showed that the addition of yeast metabolites seemed to be beneficial for the growth of all isolates, when compared to freshly diluted molasses (Supplementary Fig. 7). Potentially, glycerol, being a reduced compound, acted as a carbon source for the bacteria, allowing the population to sustain a higher final OD as compared to the remaining culture media. In addition, the fact that in depleted molasses supplemented with sugars all bacteria showed a lower OD as compared to molasses spiked with yeast metabolites indicates that competition for nutrients might be an important factor in yeast-bacteria interactions. Moreover, although the beneficial strain B showed the highest final OD values (Supplementary Fig. 8), the detrimental strain C was generally less affected by the spent yeast media under all evaluated conditions as compared to the control condition (Supplementary Fig. 7). These results suggest that this strain might be fitter for faster growth during the fermentation, when compared to other *L. fermentum* strains, and environmental conditions might play a big role in defining which dominant strain would overtake the *L. fermentum* population in the process.

### Higher temperatures may encourage growth of detrimental *L. fermentum* strains during fermentation

**Fig. 5:**
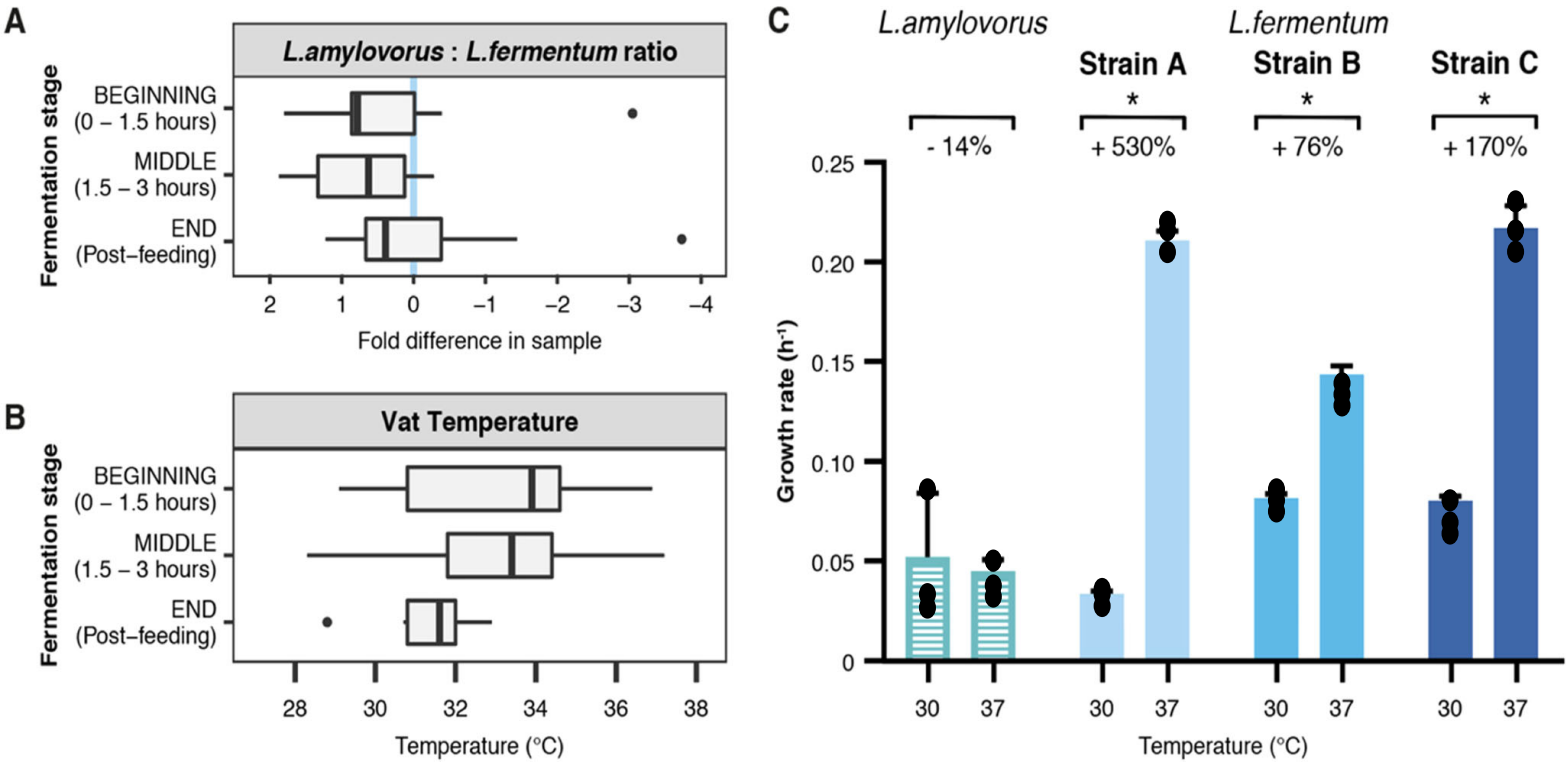
Temperature has different effects on *L. amylovorus* and *L. fermentum* growth rate during industrial fermentation. **A** *L. amylovorus*-to-*L. fermentum* ratio at each stage of fermentation, summarised for both mills. Blue line denotes equal proportion of the two bacteria (i.e. zero-fold difference). *L. fermentum* populations can overtake *L. amylovorus* at the end of the bioprocess (right of blue line). For samples containing more than one datapoint, *n* = 3 biologically independent samples. The centre line denotes the median value (50th percentile), while the box contains the 25th–75th percentiles of dataset. The whiskers mark the 5th and 95th percentiles, and values beyond these upper and lower bounds are considered outliers, marked with dots. **B** Vat temperature at each stage of fermentation, summarised for both mills. Temperature decreases as the bioprocess progresses. For samples containing more than one datapoint, *n* = 3 biologically independent samples. The centre line denotes the median value (50th percentile), while the box contains the 25th – 75th percentiles of dataset. The whiskers mark the 5th and 95th percentiles, and values beyond these upper and lower bounds are considered outliers, marked with dots. **C** Growth rates of *L. amylovorus* and *L. fermentum* isolate strains A, B and C at 30°C and 37 °C. Higher fermentation temperatures hamper *L. amylovorus* growth (left, striped) and favoured *L. fermentum* strains (blue), with increases of up to 530%. Error bars denote variation across experimental replicates (*p < 0.05). For samples containing more than one datapoint, *n* = 3 biologically independent samples. Data are presented as mean values +/- SD. Final ethanol yields were compared by multiple *t-*test (statistical significance analysis with alpha value of 0.05). Source data are provided as a Source Data file.

Our results indicated that some bacterial strains in the industrial fermentation microbiome can have beneficial effects on bioconversion efficiency. However, these are often removed from the process by the broad-spectrum methods employed by biorefineries. This motivates the need for more targeted approaches that can be practicably applied to current industrial setups to control populations of detrimental bacteria^29^. Focusing on the retention of *L. amylovorus* and reduction of *L. fermentum*, we found that the latter was more likely to flourish at the end of the fermentation process, irrespective of batch performance or vat temperature (Fig. 5A, B, Supplementary Fig. 8). As no link was found between these two parameters, we posited that the bacteria grew optimally at different temperatures. We tested this on our *L. amylovorus* and *L. fermentum* isolates by comparing their growth at 30 °C and 37 °C (Fig. 5C, Methods). The growth rate of *L. amylovorus* was found to be 14% higher at 30 °C, from 0.052 ± 0.026 to 0.045 ± 0.005 h^−1^. In contrast, all *L. fermentum* strains favoured the higher temperature, with increases as high as 5-fold (multiple *t*-test, *p* < 0.05; strain A: from 0.330 ± 0.001 to 0.211 ± 0.004 h^−1^; strain B: 0.082 ± 0.002 to 0.143 ± 0.004 h^−1^; strain C: 0.080 ± 0.002 to 0.217 ± 0.009 h^−1^). Vat temperature at the end stage of fermentation could thus act as a viable predictor of the ratio between these two key species, providing a means to reduce the impact of detrimental *L. fermentum* strains.

### Metagenome-assembled genomes analyses

For validation purpose, we implemented a metagenome-assembled genomes (MAGs) pipeline that integrates state-of-the-art tools into a streamlined, user-friendly system (Methods). The assembly performance is noteworthy, with the resulting MAGs achieving an N50 sequence length exceeding 14 kilobases (Supplementary Data 3). Subsequently, we conducted taxonomy profiling of the MAGs, which revealed *L. fermentum and L. amylovorus* as abundant bacterial species in 45 and 51 samples respectively (see last column in Supplementary Data 3). Their abundances across samples are presented in Supplementary Fig. 9A. This finding aligns with the results observed.

In this study, the gene annotations and metabolic characteristics of three Lactobacillus fermentum strains are presented, using metagenome-assembled genomes (MAGs) from various samples (Supplementary Fig. 9B, Methods). The findings indicate a high level of conservation in genes and metabolic pathways among these strains. However, two unique genes, specific to M0060 module in category l, were identified in strain IFO3956. The study also reinforces previous observations that certain *L. fermentum* strains are associated with decreased performance, demonstrating a link between specific bacterial strains and their biological impact.

## Discussion

In this study, we have used high-resolution metagenomic sequencing and physiological data from laboratory experiments, combined with in-depth surveying of two independent biorefineries, to build a comprehensive map of the microbial ecology underlying industrial-scale bioethanol production. We provide insights into how selective pressures imposed by the fermentation process shape microbiome functionality and ultimately, efficiency of the bioconversion process.

The unidirectional nature of the fermentation setup provided a unique system to study the interactions within a complex microbial community and how it changes in response to quantifiable changes in its environment. We demonstrate in vitro that higher temperatures encourage rapid growth of *L. fermentum*, which could drive the dominance of bacteria over *S. cerevisiae* populations that may already be under heat stress. This, along with increased competition for limited resources, likely reduces viability of the yeast cells. This interplay motivates the adoption of sequencing technologies, in place of rough estimations such as total cell counts, to evaluate and predict bioconversion outcome more accurately and thus improve process control of this industry. As we demonstrate in this study, our metagenomic approach also enables the discovery of microbial species and functions not previously associated with fermentation performance.

We show that bacteria not only hinder but also enhance the industrial production of bioethanol. Importantly, impact on industrial productivity is likely to be mediated by particular strains rather than entire species. This suggests that current microbial management approaches aimed at removing all bacteria from the bioconversion process may be counterproductive and in fact reduce overall ethanol yields. To this end, monitoring of the bacterial community provides strategic first steps towards improving overall performance of the industrial process. In addition to ensuring optimal fermentation activity by *S. cerevisiae*, tighter control of conditions such as vat temperature may also facilitate the retention of a microbiome that supports sustainable production of bioethanol.

Considering that the presence of specific *L. fermentum* strains can lower ethanol yields by almost 5%, the adoption of more targeted strategies that minimise the growth of these detrimental strains, while preserving beneficial *L. amylovorus* and *P. claussenii* populations, could translate to projected gains of over US$1.6 billion^30^, and a reduction of >2 million tons in carbon emissions, considering the Brazilian bioethanol industry alone^31^.

## Methods

### Chemicals

Unless stated otherwise, all chemicals and reagents used were purchased from Sigma-Aldrich (St. Louis, MO, USA).

### Sampling strategy

We sampled two independent ethanol mills (named Mill A and Mill B) in the production season of 2017. Both mills are located in the State of São Paulo, Brazil in a region with the prevalence of the humid subtropical climate (*Cfa*) with an annual precipitation of around 2000 mm, and sea-level altitude of *ca*. 600 m. The mills were completely independent from each other, with a distance >300 km apart, and have raw material sourced from different producers and sugarcane fields. Both mills operated via fed-batch fermentations (Melle-Boinot setup), performing up to 3 cycles per day, and had a similar ethanol production capacity with a daily output of ca. 400m^3^ of ethanol. Mill A was sampled on dates: 26/05/2017, 26/10/2017 and 17/11/2017. Mill B was sampled on dates: 02/06/2017, 29/10/2017 and 03/11/2017. The following steps of the ethanol production process were sampled: (1) Fermentation broth (Feeding line with fresh fermentation media); (2) start (0–1.5 h after feeding has commenced); (3) middle (1.5−3 h); (4) end of fermentation (after cessation of feeding); (5) yeast cream after separation of the wine (which is sent to distillation centrifugation); and (6) biomass after acid wash treatment (sulphuric acid pH 2.5 for 1 h). As different vessels are fed sequentially, samples were collected from different vats at different stages of fermentation in a single day. Samples were collected directly from the production process and diluted 1x in a sterile Phosphate Buffered Saline (PBS) solution with glycerol (50%). The samples were readily frozen in dry ice, until final storage in ultrafreezer (−80 °C). Each mill had several vessels operating in the same fermentation step, which allowed for process replicates. Samples were taken in duplicates.

### Industrial metadata

The industrial metadata was provided by the operational staff from each mill and consisted of key process control parameters collected and registered by industrial staff, related to the ethanol fermentation. We used the following parameters: ethanol yield, acidity from wine (g_acetic_ acid equivalent/L, where g_acetic_ acid equivalent is related to the amount, in g/L, of acetic acid equivalent obtained via titration); yeast cell counts in the fermentation (CFU); bacteria cell counts in the fermentation (CFU); yeast viability (% of the population); yeast budding rate (% of the population); vessel current volume (in m^3^) and vessel temperature (°C). These are provided in Supplementary Data 1. For correlation analyses, the data was converted into monthly averages.

### Industrial performance calculation

The industrial performance calculation was obtained by the product of the multiplication of the parameters directed correlated with process performance (i.e. ethanol yield and yeast viability), divided by the product of the multiplication of the parameters inversely correlated with process performance (i.e. bacterial cell counts and acidity titre):

1 $${Industrial\; performance}=\frac{({Ethyield} \, {x\; Yeast}{viab})}{({Baccounts} \, {x\; Acid}{titre})}$$

The score is obtained by multiplying ethanol yield (Eth_yield_) and yeast viability (Yeast_viab_) values and dividing its product by the product obtained from the multiplication of bacterial cell counts (Bac_counts_) and wine acidity titre (Acid_titre_) values.

### Strains used in laboratory experiments

*Saccharomyces cerevisiae* strain PE-2 was kindly provided by Prof. Thiago Olitta Basso (São Paulo, Brazil). Strains of *Lactobacillus amylovorus* and *Lactobacillus fermentum* were isolated from stored industrial samples. Strains of *Pediococcus claussenii*, *Lactobacillus helveticus, Lactobacillus buchneri* and *Zymomonas mobilis* were purchased from ATCC (Manassas, VA, USA).

### Isolation and maintenance of industrial strains

Industrial samples were serially diluted in sterile PBS and plated in Man Rogosa Sharpe (MRS) Agar media, containing cycloheximide (0.1% v.v^−1^) to inhibit yeast growth. Plates were incubated at either 30 °C or 37 °C statically. A loopful of an isolated colony was grown in liquid MRS in the same conditions and stored at −80 °C (see section ‘DNA extraction of bacterial isolates’ below). Yeast strains were cultured in Yeast Potato Dextrose (YPD) media, at 30 °C. Lactobacilli were cultured in MRS media, either at 30 °C or 37 °C, and *Zymomonas mobilis* was cultured at Trypsin Soy Broth (TSB) media, at 30 °C. All cultivations were performed statically, in *ca*. 5 mL volume.

### DNA extraction of bacterial isolates and metagenomic samples

Pure isolates were grown overnight in adequate media and conditions (please see section Isolation and maintenance of industrial strains). After growth, cells were pelleted via centrifugation (>10,000 g for 4 min.) and genomic DNA was extracted using the MasterPure™ Gram Positive DNA Purification Kit (Lucigen Corporation, Middleton, WI), according to manual’s instruction.

DNA extraction of metagenomic samples was performed using the DNeasy Powerlyzer Powersoil Kit (QIAGEN, Hilden, Germany), according to manufacturer instructions. Extraction was not possible for sample collected from the starting broth (Step 1) in Mill A on 17/11/2017. All DNA extraction quantifications were performed with Qubit Fluorometer (Thermo Fischer Scientific, Waltham, MS, USA).

### Sequencing of bacterial isolates and metagenomes

Shotgun metagenomics and genome sequencing of isolates were performed on the NextSeq 500 using NextSeq High Output v2 Kit (300 Cycles) (Illumina, San Diego, CA, USA) by the Sequencing Core Facility at The Novo Nordisk Foundation Center for Biosustainability (Technical University of Denmark, Kongens Lyngby, Denmark). The library preparation was performed using the KAPA HyperPlus Library Prep Kit (Roche, Basel, Switzerland), and the indexing kit used was the Dual Indexed PentAdapters, Illumina compatible (PentaBase, Odense, Denmark). Quantity and quality control were performed using Qubit dsDNA HS Assay Kit (Invitrogen, Carlsbad, CA, USA) and DNF-473 Standard Sensitivity NGS Fragment Analysis Kit (1 bp - 6000 bp; Agilent, Santa Clara, CA, USA). Average library length was 341 bp. The sequencing reads length were 150 base pair paired-end (2 × 150 bp). The index (i7 and i5) reads were 8 bp, dual indexed and flow cell loading was 1.3 pM. The sequencing chemistry used was 2-channel sequencing-by-synthesis (SBS) technology, and Phix control V3 (Illumina San Diego, CA, USA) was added (2.5%).

### Pre-processing of genomic and metagenomic data

Raw reads underwent quality trimming, i.e. filtering of adaptor and universal primer sequences, as well as low quality bases (<Q20), reads <75 bp and duplicated reads (bwa mapping against GRCh37/hg19 reference genome using mem algorithm, extracting reads with >95% identity)^32^. More information can be found in Supplementary Data 2 and Supplementary Data 3.

### Bacterial isolate assembly

Both reference-guided and de novo genome assembly were performed on the quality-filtered reads for each isolate. SPAdes 3.12^33^ was run using the following parameters: -m 300 -k 33,55,77,99,127. Reference-assisted genome assembly was performed with idba_hybrid (v 1.1.1)^34^ with the following parameters: --pre_correction --mink 120 --maxk 180 --step 10 --min_contig 300 --reference [NCBI ref genome]. Two modifications were made in the source code before compiling IDBA_UD: (1) in file src/basic/kmer.h, kNumUint64 was changed from 4–8 to allow maximum kmer length beyond 124; (2) in file src/sequence/short_sequence.h, kMaxShortSequence was set to 512 to support longer read length. Assembly statistics are provided in Supplementary Data.

### Metagenome co-assembly

To account for a high number of *S. cerevisiae* in our samples, as well as fluctuations in the microbial community as it progresses through the production process, we separated the reads into two groups – *S. cerevisiae* (SC reads) and others (non-SC reads)—and co-assembled them separately. To do this, read alignment was performed against all *S. cerevisiae* genomes (481 in total; NCBI Genome, downloaded August 2018) in concatenated form, using the BWA mem model with default parameters^32^. Reads over 95% identity were classified as SC reads; the remainder were deemed non-SC reads. Each set of reads were concatenated separately from sequenced samples, and maximum *k*-mer depth was normalised to 100-fold using BBnorm (https://sourceforge.net/projects/bbmap). Co-assembly was done by IDBA_ud (version 1.1.1)^34^, using the following parameters: -min_contig 300 –mink 50 –maxk 124 –step 10 –pre_correction. This yielded a total of 241,214 contigs, totalling 254.8 M bp. Assembly statistics are provided in Supplementary Data 2 and Supplementary Data 3.

### Gene catalogue of the bioethanol production microbiome

MetaGeneMark v3.26^35^ (using the default parameters) was used to predict coding DNA sequence (CDS) regions in the assembled metagenome contigs (both SC and non-SC). These 356,115 sequences were then clustered at 95% nucleotide identity using CD-HIT^36^. The longest DNA sequence for each cluster was used to generate the resulting catalogue of 297,115 non-redundant gene sequences with median length 336 bp.

### Taxonomic profiling of microbial communities

To profile the microbial community (encompassing eukaryotes, bacteria and archaea), the assembled metagenome contigs were processed using ‘ssu_finder’ tool in the CheckM package (version 1.0.18), which identifies small-subunit ribosomal RNA genes (i.e. 16 S rRNA in prokaryotes, 18 S rRNA in eukaryotes). The resulting 337 DNA sequences were clustered by 99% sequence identity using CD-HIT^36^. The longest sequences of each of the 151 resulting clusters were selected as representatives and classified using the SINA tool (version 1.2.11) from the SILVA rRNA database project^37^. In cases where classification was ambiguous, we used the least-common-ancestor derived from SILVA (lca_tax_slv column). One sequence classified as Plantae was excluded from the analysis. Sequences deemed “Unclassified” were further curated using EZBioCloud^38^ (version 20201012). Further information is provided in Supplementary Data 4 and 5.

Each metagenomic sample was profiled by mapping its reads to the 150 SSU sequences, using NGLess^39^ (version 1.1). Profiling of bacterial communities was done using Kraken v0.10.5-beta with default settings against the minikraken 2017.10.18 8GB database^40^. Bracken^41^ v2.0 was used for accurate species abundance estimation with parameters -r 150 -l S. Where applicable, species names have been edited in the main text to reflect updates in taxonomic classification.

### Functional analyses of bacterial isolates and metagenomes

The gene catalogue was annotated using eggNOG mapper (version 1.0.3-35-g63c274b, emapper DB: 2.0) with default settings^42–44^. Reads from each metagenomic sample were mapped to the gene catalogue and relative abundances summed by KEGG Module IDs^44^. This was done using NGLess version 1.1^39^.

Assembled genomes of the 3 *L. fermentum* isolate strains were annotated using Prokka (version 1.14.0)^45–47^. Functional characterisation was done using eggNOG-mapper^48^ (as above), ResFinder and antiSMASH (web services; February 2022) on default parameters^49–51^. Gene content of the 3 *L. fermentum* strains was compared using their gene name annotations as provided by Prokka.

### Phylogenomic analysis of *L. fermentum* strains

In addition to the 3 sequenced isolates, we used 22 *L. fermentum* genomes deemed high-quality by the proGenomes^52^ database and available in NCBI RefSeq (Supplementary Table 3; specI_v3_Cluster1407)^53^. To infer phylogenomic relationships between the strains, the 41 taxonomic marker genes were extracted from each genome using the ‘Genome Classifier’ tool^54^ and aligned using t_coffee (-method t_coffee_msa, clustalw_msa)^55^ with painfully manual corrections. The aligned sequences for each of the 25 strains were concatenated using SequenceMatrix^56^, with ‘N’ of 150 bp length inserted to separate each sequence. The combined multiple sequence alignment (MSA) was used to test for goodness-of-fit to all nucleotide substitution models available on MEGA X^57^. The GTR + G + I model (General Time Reversible model with Gamma-distributed rate variation among sites, with proportion of invariant sites) had the lowest Bayesian Information Criterion (BIC) score and was used to compute tree topology. The maximum likelihood tree was generated from 500 bootstrap replications. These analyses were also conducted using MEGA X. Habitat information for the 22 published genomes was compiled from Maistrenko et al.^58^ as well as NCBI and GOLD^59^ databases.

### Fermentation experiments

Fermentations were performed in 96 deep-well plates, with either pairwise cultivations (yeast:bacteria at a 100:1 ratio)^17^, or standalone yeast or bacteria cultivations. The media used is a semi-synthetic media, able to simulate sugarcane molasses based media (SM)^26^. Briefly, all strains were cultured in their optimal media and conditions (see above “Strains” and “Isolation of industrial strains and maintenance” sections), for up to 48 h. After that, the biomass was calculated via optical density (OD; 600 nm wavelength). All cells were pelleted via centrifugation (3400 × g, 4 °C, 15 min) and washed twice with sterile PBS. Subsequently, cells were diluted in SM diluted in sterile Milli-Q H2O (10x, final sugar concentration of 18 g/L) for an OD value of 1.0. Strains were later diluted in fresh SM media in specific wells in the 96 deep-well plate to a final OD value of 0.1.

The lactobacilli growth rate analysis was performed at 30 and 37^o^C, under agitation (double orbital, fast mode) in Synergy H1 plate readers (Biotek Instruments, Inc. Winooski, VT, USA). OD was measured every 30 min for 24 h. Growth rate was calculated using the R package *growthcurver*^60^.

All the pairwise cultivations were performed statically, overnight, at 30 °C, in ca. 1 mL volume. The fermentations were performed in triplicate. The carbohydrate titre and composition (sucrose, glucose and fructose) and fermentation metabolites (glycerol, ethanol, and acetic acid) were determined by high-performance liquid chromatography (HPLC) (UltiMate 3000, Thermo-Fischer Scientific, Waltham, Massachusetts, USA). The analites were separated using an Aminex HPX-87H ion exclusion column (Bio-Rad, Hercules, California, USA) and were isocratically eluted at 30 °C, with a flow rate of 0.6 mL/min, using a 5 mM sulphuric acid solution as mobile phase. The detection was performed refractrometrically.

Ethanol yield was calculated according using the following equation:

2 $$\,{Ethanol\; yield}=\frac{({EtOHobs} \, x\,100)}{{EtOH}{theor}}$$

EtOH_obs_: the observed ethanol titre on each sample. EtOH_theor_: the maximum theoretical ethanol titre for each sample. Obtained by multiplying the sugar titre from the broth solution with the stoichiometric conversion factor for ethanol production (i.e. 0.5111)^61^.

Community composition was resolved via flow-cytometry (BD LSRFortessa™, BD Biosciences, Franklin Lakes, New Jersey, USA). A sample from each well (10 µL) was taken after the overnight cultivation, and was transferred to a new microplate and diluted in 190 µL PBS buffer (pH 7.4). Yeast and bacteria populations were resolved via front and side scatter comparison (SSC versus FSC; Supplementary Fig. 10).

### Metabolite profiling

Lactobacilli supernatant metabolite profile was analysed via HPLC after 48 h of growth (please see section Fermentation experiments for a detailed description of the HPLC method). A pre-inoculum of lactobacilli stored at −80 °C was grown in MRS for 24 h. After that the OD from these cultures was measured and fresh MRS media was inoculated with a fixed OD of 0.1 and incubated statically at 37 °C. After growth, the cells were separated via centrifugation and the supernatant was sent for further analysis.

### Cross-feeding/metabolite inhibition experiments

To investigate the impact of lactic acid bacteria metabolites on the growth of *S. cerevisiae* PE-2, three LAB strains of *L. fermentum* (A, B, and C) were cultivated in diluted molasses (20 g l^−1^ TRS) with an initial inoculation OD of 0.5. After 48 h cultivation, the spent growth media was collected by centrifugation and filtered through a 0.22 µm filter to produce the media used for the microplate assay (referred to as “diluted molasses previously fermented by each of the *L. fermentum* strains”). The growth of *S. cerevisiae* PE-2 was evaluated using a Tecan Infinite® 200 PRO microplate reader at a temperature of 30 °C for 24 h with an initial OD of 0.1 in 200 µL of the following media: (1) diluted molasses (20 g l^−1^ TRS), (2) diluted molasses previously fermented by each of the *L. fermentum* strains; (3) diluted molasses previously fermented by each of the *L. fermentum* strains supplemented with a mixture of sugars (to restore its initial sugar composition) and (4) diluted molasses spiked with key bacterial metabolites (i.e., acetic and lactic acids), to restore the composition produced in the diluted molasses previously fermented by each of the *L. fermentum* strains.

Similarly, to investigate the impact of yeast growth metabolites on lactic acid bacteria growth, *S. cerevisiae* PE-2 was cultivated in diluted molasses (20 g l^−1^ TRS) with an initial inoculation OD of 0.5. After 8 h, the spent growth media was collected by centrifugation and filtered through a 0.22 µm filter to produce the media used for the microplate assay (referred to as “diluted molasses previously fermented by *S. cerevisiae*”). The growth of each *L. fermentum* strain (A, B, and C) was evaluated using a Tecan Infinite® 200 PRO microplate reader at a temperature of 30°C for 150 h with an initial OD of 0.1 in 200 µL of the following media: (1) diluted molasses (20 g l^−1^ TRS), (2) diluted molasses previously fermented by *S. cerevisiae*; (3) diluted molasses previously fermented by *S. cerevisiae* supplemented with a mixture of sugars (to restore its initial sugar composition) and (4) diluted molasses spiked with key yeast metabolites (i.e., glycerol and ethanol), to restore the composition produced in the diluted molasses previously fermented by *S. cerevisiae*.

From the growth profiles, specific growth rate (μ) and final OD values were calculated and Tukey’s Multiple Comparison Test was performed with *p* < 0.05 to evaluate the statistically significant difference between the treatments. The media composition utilized in the microplate experiments can be found in the Supplementary Tables 4 and 5.

The industrial molasses sample used in this study was obtained from an ethanol production plant and was diluted in distilled water to a concentration of 20 g L^−1^ TRS and sterilised through a 0.22 µm filter. The final sugar concentration was determined using an ion exchange column HPX-87C (Bio-Rad) at 85 °C with H_2_O as the mobile phase and a flow rate of 0.6 l min^−1^ for glucose, fructose, and sucrose. The microbial metabolites concentrations were obtained using an ion exchange column HPX-87H (Bio-Rad) at 60 °C with 5 mM H_2_SO_4_ as the mobile phase and a flow rate of 0.6 l min^−1^ for lactic acid, acetic acid, glycerol, and ethanol.

### Statistical analyses

Rarefaction of read counts and subsequent analyses were done using R and packages *vegan*^62^ and *tidyverse*^63^. Community compositions were compared using Bray-Curtis distance on species relative abundance and Permutational Multivariate Analysis of Variance (PERMANOVA) with 999 permutations and the Bray-Curtis method was applied by providing Mill/Process step/Date as function. Spearman’s correlation coefficient was calculated for each pair of industrial metadata variables, and between metadata variables and species abundances. False discovery rate (FDR) was calculated using Benjamini-Hochberg (BH) method, with FDR < 0.1 used as cut-off. Statistical analyses for fermentation experiments were performed using the software GraphPad Prism 8. Final ethanol yields were compared by multiple *t-*test (statistical significance analysis with alpha value of 0.05).

### Metagenome-assembled genomes pipeline

In this section, we present the comprehensive and detailed methodology for the analysis of metagenomic raw data^64^, addressing every aspect from initial quality control to the final annotation and classification stages/MAG production:

1. Quality control and trimming: The process starts with paired end reads of 150 bp. FastQc (v0.12.1) inspects the raw reads for quality control, providing a preliminary assessment of potential issues in the sequence data. Subsequent trimming is performed using bbduk (v39.00), a kmer-based decontamination tool. This step involves the removal of adaptor sequences and the exclusion of sequences with a Phred score <33 and a minimum read length of 100 bp. The parameters set for this process include qin = 33 for input quality offset, hdist = 1 for Hamming distance in error correction, and trimq = 30 for quality trimming, among others.

2. Assembly and dereplication: The initial co-assembly of the trimmed reads is executed with MEGAHIT (v1.2.9)^65^ for every sample, which assembles by incrementing odd-number kmers to avoid palindromes. It’s important to note that we opted for a per-sample assembly approach with MEGAHIT to ensure specificity and to accurately reflect the diversity inherent in different fermentation processes and mills. The subsequent dereplication step involves CD-HIT-EST (v4.8.1), where contigs with a minimum of 99% global sequence similarity are combined to eliminate redundant sequences. A secondary assembly is then performed with CAP3, focusing on contigs with an identity and overlap length cutoff of ≥97%^66^.

3. Contig quality control: After assembly, the contigs undergo quality control using Quast.

4. Coverage analysis and binning: The sequencing depth or coverage is determined by aligning the short reads to the co-assembled contigs using BWA MEM (v0.7.17-r1188) and further processed with Samtools (v1.16.1) and Bedtools (v2.31.0). This data is then utilized in the binning process, employing state-of-the-art tools like MetaBAT2, MaxBin2, and CONCOCT^65^. These tools are chosen to minimize biases and maximize accuracy, with each providing a unique approach to binning. The outputs from these tools are aggregated using DASTool (v1.1.6), which refines the bins by dereplicating contigs and selecting high-quality candidates.

5. Quality check of binned contigs: The quality of the binned contigs is assessed using CheckM (v1.2.2) with its “lineage wf” workflow. This step is crucial for evaluating the completeness and contamination levels of the binned contigs. Meta-QUAST is also employed for additional quality control^67^.

### Functional annotation and taxonomical identification of MAGs

For functional annotation, tools like CAT/BAT, PROKKA, and eggNOG are used, with Prodigal (v2.6.3) serving as the protein predictor. The predicted proteins are compared against the NCBI nonredundant database using DIAMOND (v2.1.8.162). Taxonomical identification is performed using KRAKEN2 (v2.1.3), a kmer-based method for fast and accurate classification, using its standard database^68^.

### Reporting summary

Further information on research design is available in the Nature Portfolio Reporting Summary linked to this article.

### Supplementary information

Supplementary Information

Peer Review File

Description of Additional Supplementary Files

Supplementary Data 1

Supplementary Data 2

Supplementary Data 3

Supplementary Data 4

Supplementary Data 5

Reporting Summary

### Source data

Source Data

## Data Availability

Metagenomic sequence data are deposited in the European Nucleotide Archive under accession number PRJEB33675. Assembled genomes are under accession PRJEB52385. The gene catalogue created in this study is available via the Zenodo data archive [https://zenodo.org/records/11230028]. Source data are provided with this paper.
