# Supplementary material for: Strain dynamics of contaminating bacteria modulate the yield of ethanol biorefineries
Source: Nat Commun. 2024 Jun 22;15:5323. doi: 10.1038/s41467-024-49683-2 (PMC11193817; doi:10.1038/s41467-024-49683-2)
Supplement: Supplementary file 1 — Supplementary Information [file 41467_2024_49683_MOESM1_ESM.pdf]

Strain dynamics of contaminating bacteria modulate the yield of ethanol biorefineries

Lino *et al.*

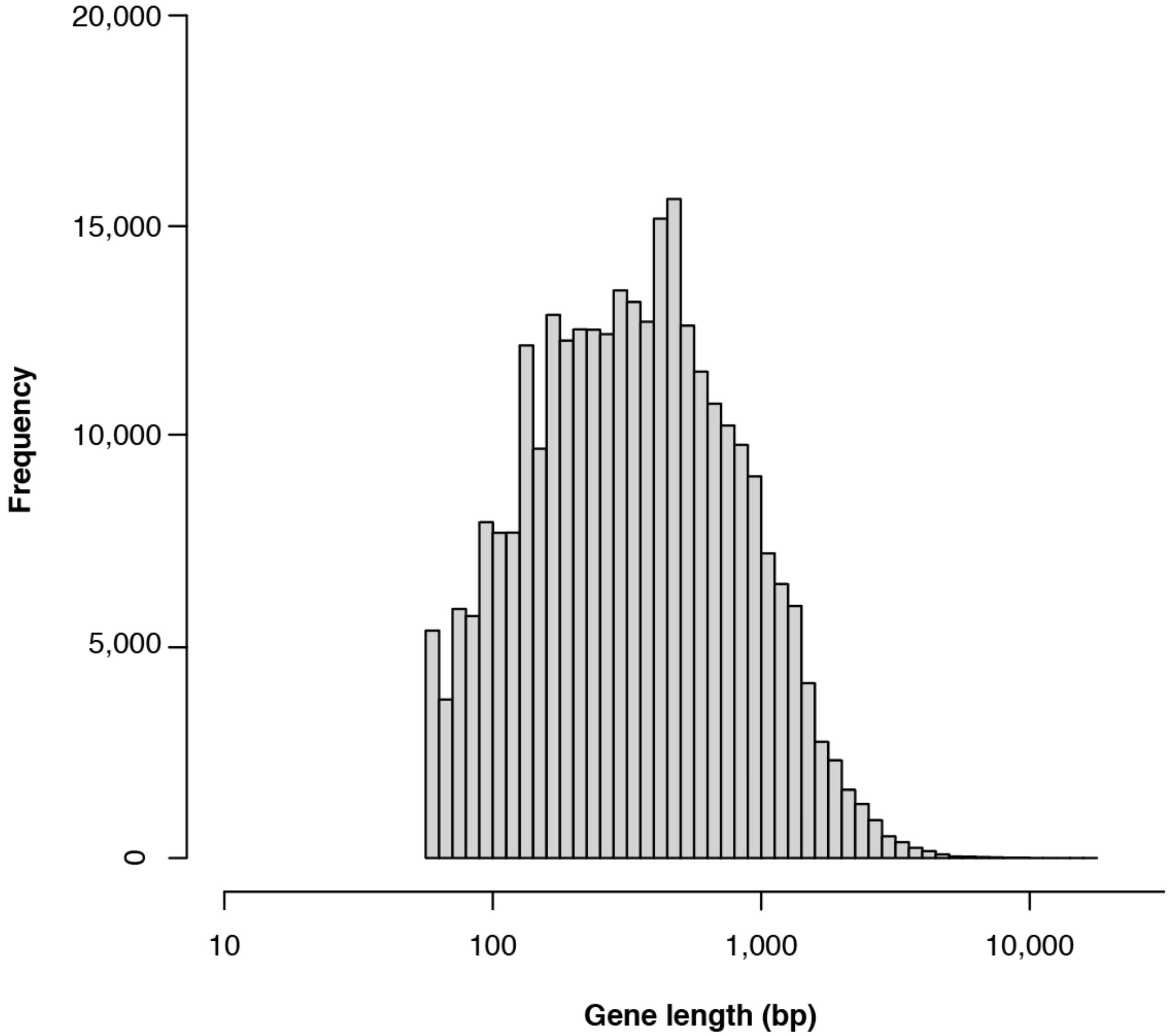

Supplementary Figure 1. Distribution of sequence lengths in the bioethanol production gene catalogue. Gene lengths ranged from 57 – 17,469 bp, with median of 336 bp. Source data are provided as a Source Data file.

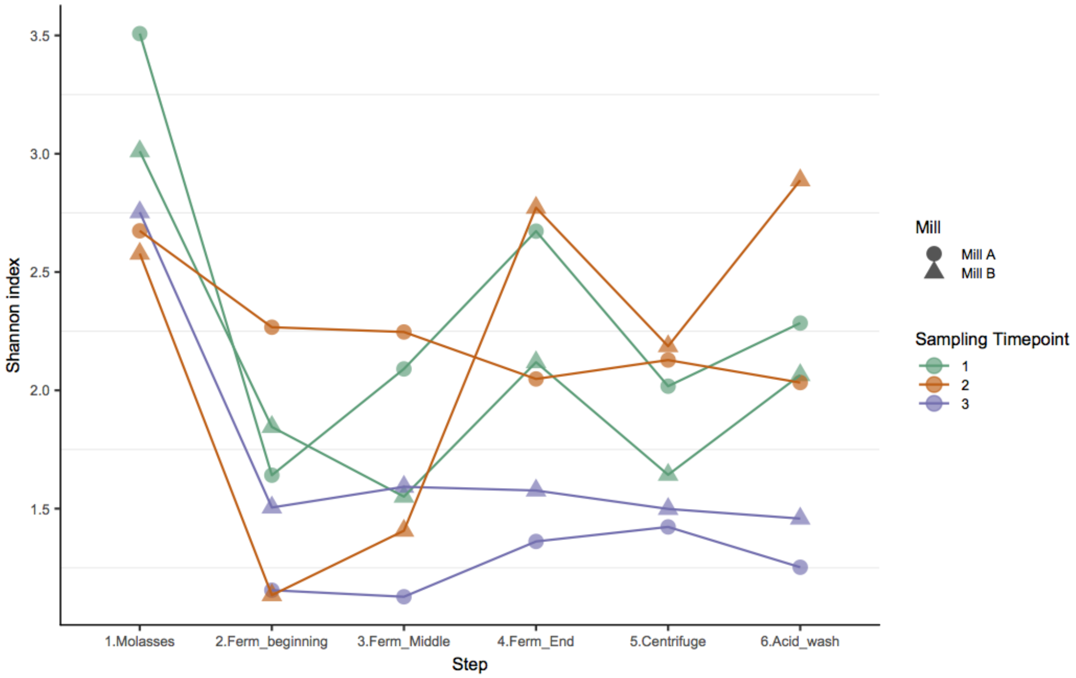

Supplementary Figure 2. Compositional changes within the bacterial community, across process steps in a single production season. Comparison of Shannon diversity index values for each collected sample: 1) molasses (or broth); 2) beginning, 3) middle, and 4) end of fermentation; 5) centrifuge; and 6) acid wash, in Mills A (circle) and B (triangle). Samples from the same collection timepoint share colour; line connects those from the same mill. Fermentation drastically reduces bacterial diversity in the starting molasses, which recovers as the bioprocess continues. Community diversity remains relatively stable in samples collected at the end of the production season (purple). Source data are provided as a Source Data file.

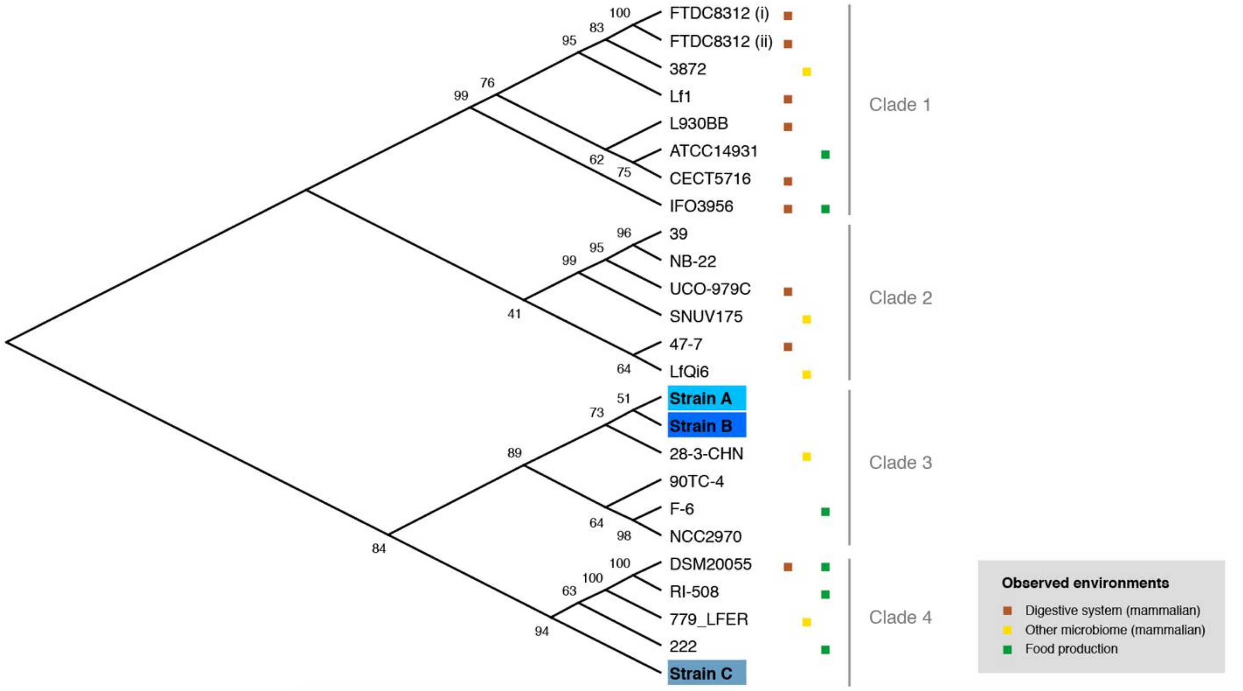

Supplementary Figure 3. Strains A, B and C cluster with *L. fermentum* strains isolated from other industrial processes. Cladogram is identical to Fig. 4B in the manuscript (here, with no adjustments to the root). Each strain is marked with coloured boxes denoting the environments it has been observed in (from other studies). While clades 1 and 2 primarily consist of *L. fermentum* strains associated with the mammalian gut microbiome, strains in clades 3 and 4 (containing our isolates A and B, and C, respectively) originate from an industrial setting, e.g. food production. No information was available for *L. fermentum* strains 39, NB-22, 90TC-4 and NCC2970. Source data are provided as a Source Data file.

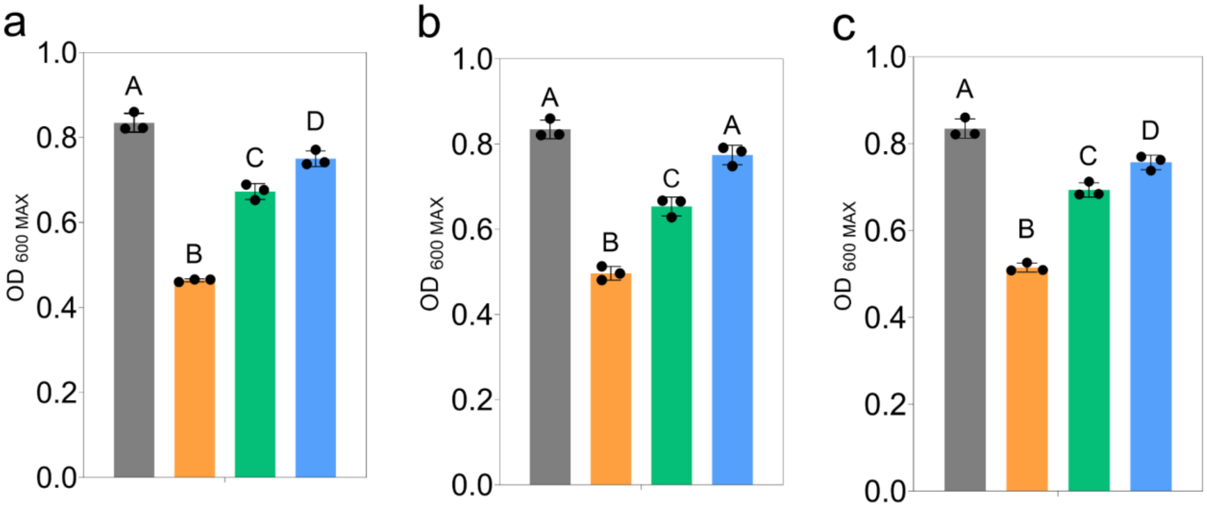

Supplementary Figure 4. Statistical comparison of maximum optical density (OD600) of *Saccharomyces cerevisiae*. OD600 observed for each *L. fermentum* a) strain A, b) strain B and c) strain C in 4 conditions: diluted sugarcane molasses (gray), molasses previously fermented with *L. fermentum* strain (orange), molasses previously fermented with added sugar (to restore the original sugar titres) (green), fresh molasses spiked with key bacterial metabolites (blue). In each graph, treatments sharing the same letter have no significant difference in maximum optical density and treatments with different letters have significant difference in maximum optical density. Source data are provided as a Source Data file.

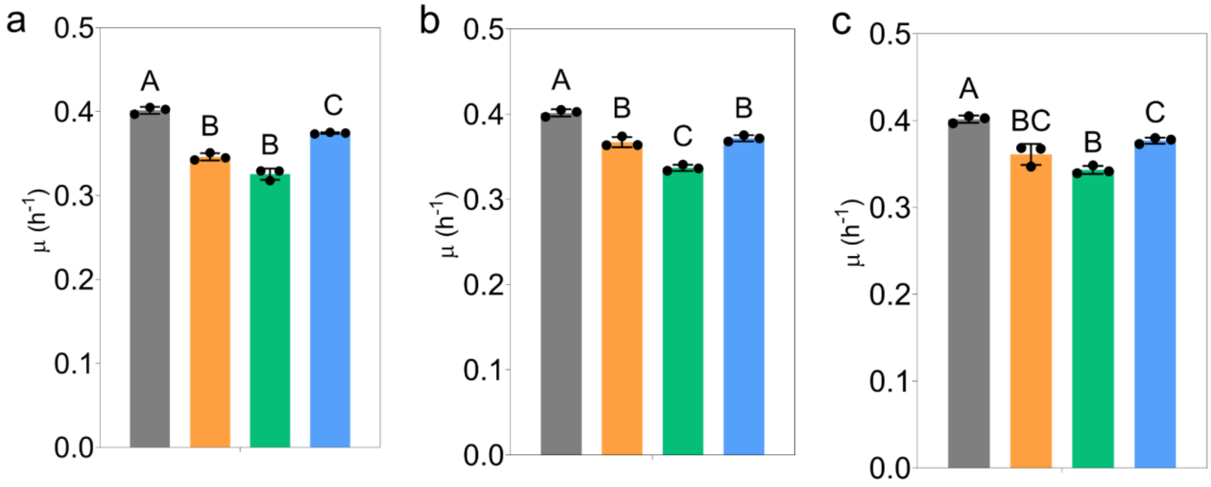

Supplementary Figure 5. Statistical comparison of maximum specific growth rates of *Saccharomyces cerevisiae*. Specific growth rates observed for each *L. fermentum* a) Strain A, b) Strain B and c) Strain C in 4 conditions: diluted sugarcane molasses (gray), molasses previously fermented with *L. fermentum* strain (orange), molasses previously fermented with added sugar (to restore the original sugar titres) (green), fresh molasses spiked with key bacterial metabolites (blue). In each graph, treatments sharing the same letter have no significant difference in maximum specific growth rate and treatments with different letters have significant difference in maximum specific growth rate. Tukey's multiple comparison test - $P < 0.05$. Source data are provided as a Source Data file.

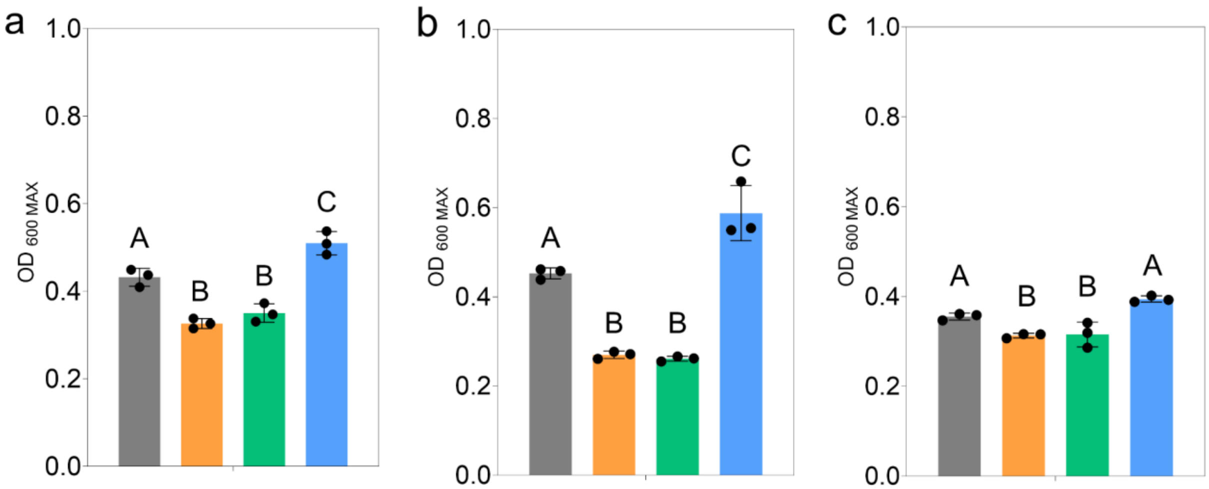

Supplementary Figure 6. Statistical comparison of maximum optical density (OD600) of each *L. fermentum* strain. a) Strain A, b) Strain B and c) Strain C observed for in 4 conditions: diluted sugarcane molasses (gray), molasses previously fermented with *S. cerevisiae* strain (orange), molasses previously fermented with added sugar (to restore the original sugar titres) (green), fresh molasses spiked with key yeast metabolites (blue). In each graph, treatments sharing the same letter have no significant difference in maximum optical density (OD600) and treatments with different letters have significant difference in maximum optical density (OD600). Tukey's multiple comparison test - $P < 0.05$

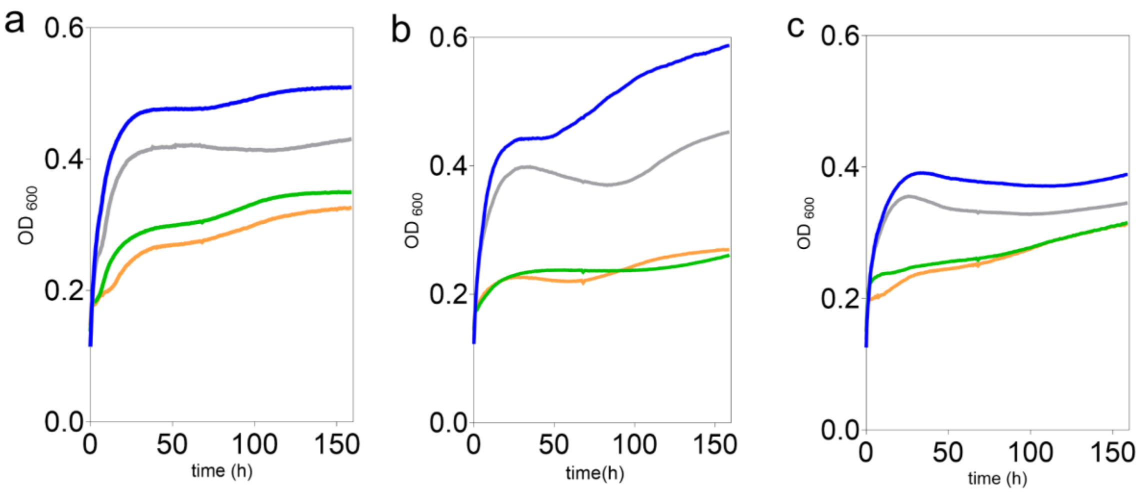

Supplementary Figure 7. Growth curve of *L. fermentum.* a) Strain A, b) Strain B and c) Strain C. Growth curve obtained in the following media: diluted sugarcane molasses (gray), molasses previously fermented with *S. cerevisiae* strain (orange), molasses previously fermented with added sugar (to restore the original sugar titres) (green), fresh molasses spiked with key yeast metabolites (blue). Growth conducted in triplicate on the Tecan Infinite® 200 PRO microplate reader at a temperature of 30°C for 150 hours with an initial OD of 0.1

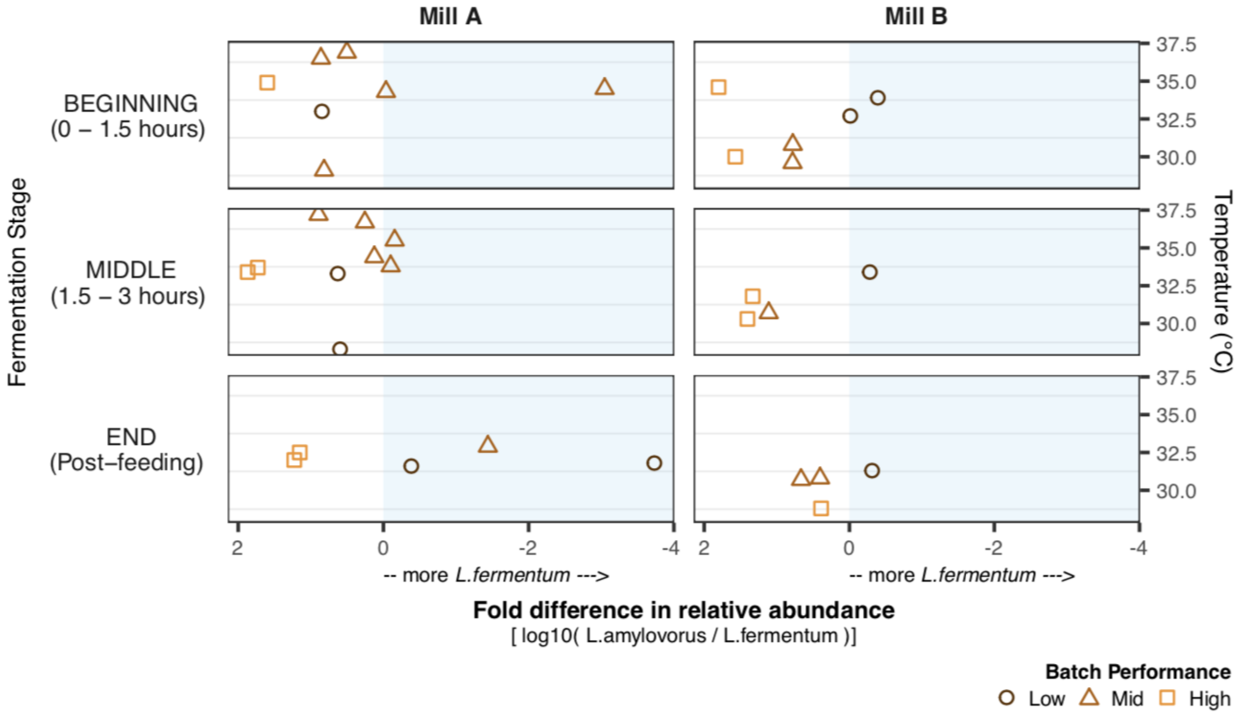

Supplementary Figure 8. Temperature does not influence *L. fermentum* and *L. amylovorus* interplay or batch performance. Comparison of temperature and *L. fermentum* and *L. amylovorus* populations on fermentation performance in Mills A and B (columns). Zero-fold difference denotes equal amount of the two bacteria; blue region highlights batches with greater numbers of *L. fermentum*. In both mills, batches displaying poorer performance are characterised by a dominance of *L. fermentum* over *L. amylovorus* at the final fermentation step, after media feeding has stopped (circles and triangles in the blue region, bottom row). Fermentation performance is not directly impacted by vat temperature.

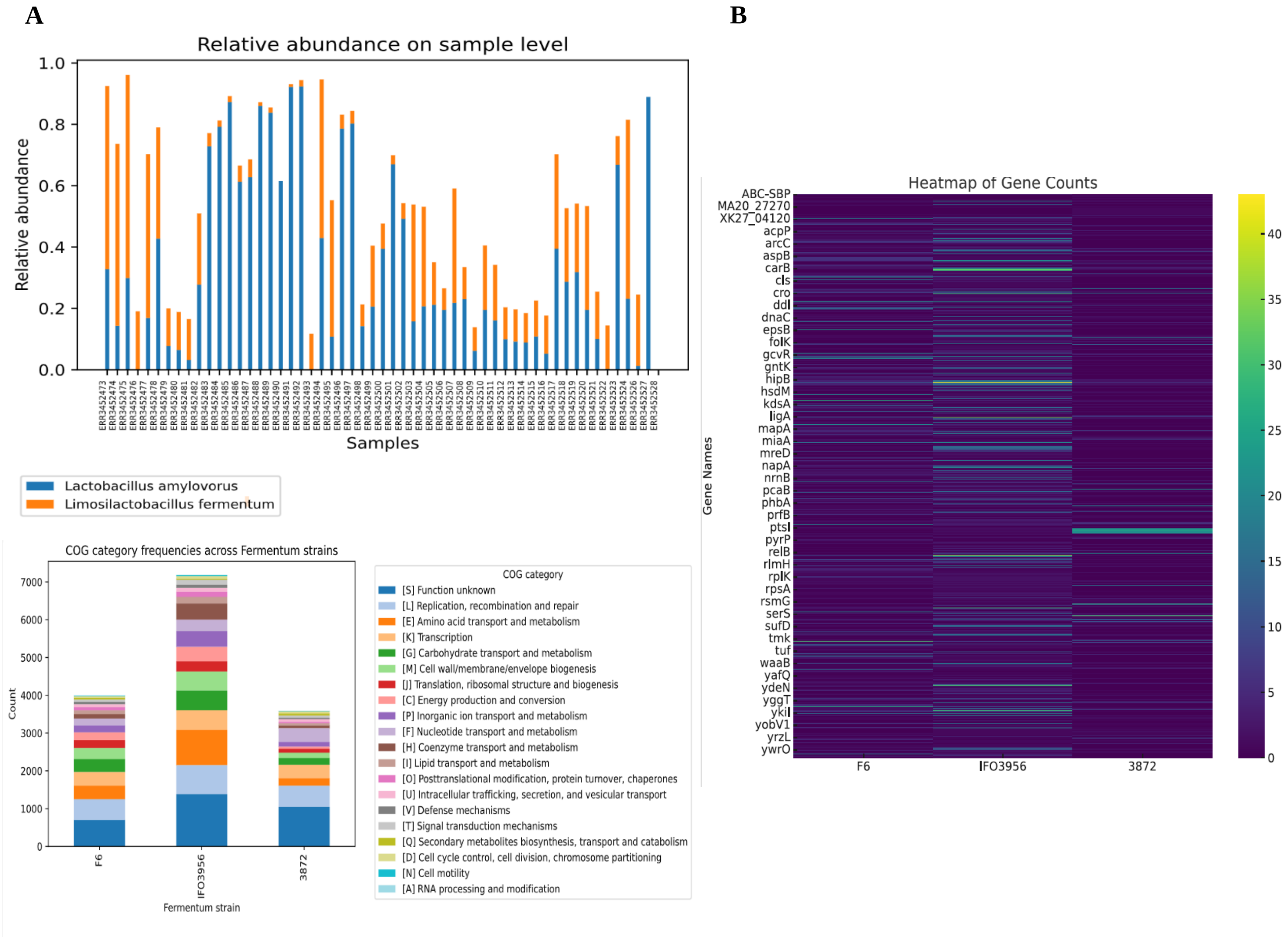

Supplementary Figure 9. Metagenome-assembled genomes (MAGs) analyses. A: Relative bacterial abundance values obtained from MAGs for *L.fermentum and L.amylovorus* across all samples. B: Using MAGs from all samples, gene annotations of three *L.fermentum* strains along with their metabolic characterisation: (top) COG category frequencies across strains; (bottom) Heatmap of gene annotation counts across strains. Source data are provided as a Source Data file.

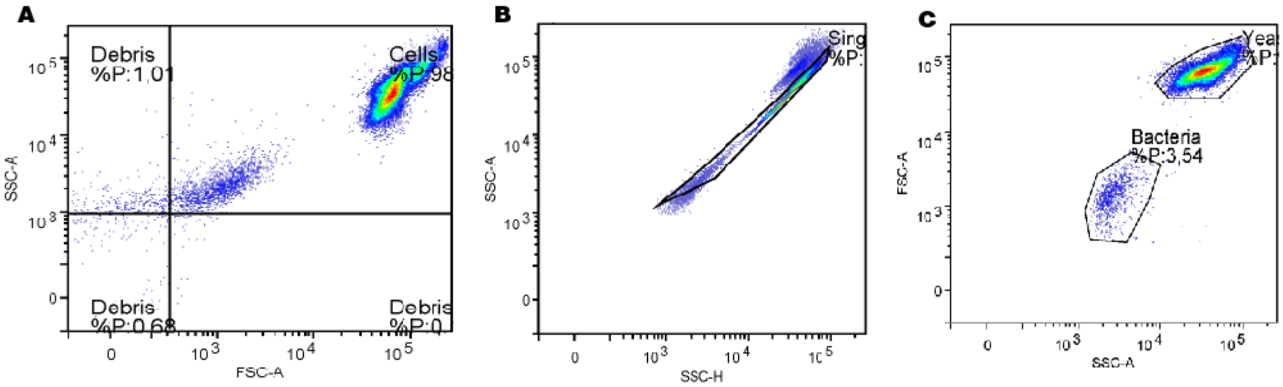

Supplementary Figure 10. The gating strategy applied to resolve both yeast and bacterial populations. A: Initially cells are separated from media debris, using side scatter (SSC-A) and front scatter (FSC-A) parameters. The gating named "Cells" contain all the yeast and bacteria cells, and not the media debris. B: By comparing area versus height parameters (SSC-A and SSC-H, respectively) from the gated events from the gate "Cells", the singlets are separated from duplets, in the gate "Singlets". C: Yeast and bacterial cells are separated based on their volume and granularity, using SSC-A and FSC-A, respectively, from the gate "Singlets". Source data are provided as a Source Data file.

**Supplementary Table 1. Taxonomic profiling of bacterial populations across study samples.**						
**Species**	**Phylum**	**Relative abundance (average %)**	**st.dev.**	**Relative abundance (minimum %)**	**Relative abundance (maximum %)**	**Samples detected (of 56)**
Lactobacillus amylovorus	Firmicutes	45,2	24,8	0,008	76,3	56
Lactobacillus fermentum	Firmicutes	19,6	17,6	0,945	60,5	56
Lactobacillus helveticus	Firmicutes	5,3	3,6	0,029	16,2	56
Pediococcus claussenii	Firmicutes	4,7	2,0	0,148	8,0	56
Lactobacillus buchneri	Firmicutes	2,0	5,1	0,048	33,0	56
Zymomonas mobilis	Proteobacteria	2,0	4,3	0,005	26,6	55
Lactobacillus plantarum	Firmicutes	1,5	1,9	0,121	12,7	55
Lactobacillus mucosae	Firmicutes	1,4	1,7	0,032	6,3	55
Acetobacter pasteurianus	Proteobacteria	1,3	7,6	0,001	56,9	51
Bacillus cereus	Firmicutes	1,2	2,8	0,003	18,6	56
Leuconostoc mesenteroides	Firmicutes	1,1	4,1	0,03	29,8	56
Streptococcus infantarius	Firmicutes	0,9	1,8	0,002	11,8	56
Lactobacillus paracasei	Firmicutes	0,9	1,0	0,061	5,3	55
Lactobacillus amylolyticus	Firmicutes	0,8	1,4	0,002	7,1	56
Lactobacillus gallinarum	Firmicutes	0,7	0,4	0,011	2,1	56
Lactobacillus rhamnosus	Firmicutes	0,6	1,5	0,008	6,6	56
Geobacillus stearothermophilus	Firmicutes	0,5	1,1	0,001	5,5	41
Lactobacillus casei	Firmicutes	0,4	0,3	0,01	2,2	56
Lactobacillus parabuchneri	Firmicutes	0,4	0,5	0,008	2,3	56
Bacillus coagulans	Firmicutes	0,4	0,7	0,001	3,0	52
Geobacillus lituanicus	Firmicutes	0,4	0,8	0,006	4,0	37
Lactobacillus delbrueckii	Firmicutes	0,3	0,5	0,048	2,5	56
Parageobacillus thermoglucosidar	Firmicutes	0,3	0,7	0,003	2,9	35
Geobacillus thermoleovorans	Firmicutes	0,3	0,6	0,005	3,2	34
Lactobacillus acetotolerans	Firmicutes	0,2	0,4	0,004	2,4	56
Acetobacter senegalensis	Proteobacteria	0,2	0,8	0,001	5,1	36
Weissella paramesenteroides	Firmicutes	0,2	0,4	0,003	1,9	56
Geobacillus kaustophilus	Firmicutes	0,2	0,3	0,002	1,8	32
Acetobacter aceti	Proteobacteria	0,2	1,1	0,001	8,2	24
Lactobacillus agilis	Firmicutes	0,1	0,3	0,001	1,5	52
Cutibacterium acnes	Actinobacteria	0,1	0,3	0,001	1,8	49
Weissella cibaria	Firmicutes	0,1	0,3	0,008	2,2	56
Escherichia coli	Proteobacteria	0,1	0,2	0,001	1,3	51
Stenotrophomonas maltophilia	Proteobacteria	0,1	0,3	0,001	2,6	34
Mycobacterium tuberculosis	Actinobacteria	0,0	0,3	0,001	2,3	56
Gluconobacter oxydans	Proteobacteria	0,0	0,2	0,001	1,4	35
Leuconostoc citreum	Firmicutes	0,0	0,2	0,001	1,6	50
Enterobacter cloacae	Proteobacteria	0,0	0,2	0,001	1,1	53
Pantoea ananatis	Proteobacteria	0,0	0,2	0,001	1,7	29
Lactobacillus pentosus	Firmicutes	0,0	0,2	0,001	1,3	50
Xanthomonas albilineans	Proteobacteria	0,0	0,1	0,001	1,1	33
Burkholderia gladioli	Proteobacteria	0,0	0,2	0,001	1,3	27
Gluconacetobacter diazotrophicus	Proteobacteria	0,0	0,2	0,001	1,1	31
Pseudomonas tolaasii	Proteobacteria	0,0	0,2	0,001	1,2	27
Cupriavidus metallidurans	Proteobacteria	0,0	0,2	0,001	1,3	11
Methylobacterium radiotolerans	Proteobacteria	0,0	0,2	0,002	1,2	13
Enterobacter asburiae	Proteobacteria	0,0	0,1	0,001	1,1	23
Mycobacterium sp. MS1601	Actinobacteria	0,0	0,2	0,001	1,2	21

Top 10 most abundant species

Supplementary Table 2. Yeast and bacteria cell counts in pairwise cultivations.

**Pairwise cultivation/ yeast bacteria ratio**	**Change in yeast population (% against control)**	**Change in bacteria population (% against control)**
PE-2/*L. fermentum* strain A/100:1	-7,4	-94,8
PE-2/*L. fermentum* strain B/100:1	-6,5	-93,9
PE-2/*L. fermentum* strain C/100:1	-23,5	-93,4
PE-2/*L. amylovorus*/100:1	1	-48,9
PE-2/*L. helveticus*/100:1	-1,4	-84,2
PE-2/*P. claussenii*/100:1	-5	-85,3
PE-2/*L. buchneri*/100:1	-2	-93,5
PE-2/*Z. mobilis*/100:1	-3	-96,3

Control: Standalone yeast and bacteria cultivations.

Supplementary Table 3. ***L.fermentum*** **strains used in phylogenomic analysis.**

Clade	Strain name	Name used	NCBI BioSample ID	Origin
1	FTDC8312	FTDC8312 (i)	SAMN02469912	Human digestive tract
	FTDC 8312	FTDC8312 (ii)	SAMN06703219	Human digestive tract
	3872	-	SAMN02314197	Human breastmilk
	Lf1	-	SAMN02053534	Human digestive tract
	L930BB	-	SAMEA3158477	Human digestive tract
	ATCC 14931	ATCC14931	SAMN00001473	Human vaginal tract
	CECT 5716	CECT5716	SAMN02604100	Human digestive tract; Human oral cavity
	IFO 3956	IFO3956	SAMD00060917	Human digestive tract; Bird digestive tract
2	39	-	SAMN03452287	
	NB-22	-	SAMN02470787	
	UCO-979C	-	SAMN04100088	Human digestive tract
	SNUV175	-	SAMN06174220	Human vaginal tract
	47-7	-	SAMN05893390	Human digestive tract
	LfQi6	-	SAMN03372370	Human breastmilk
3	28-3-CHN	-	SAMN02463745	Human vaginal tract
	90 TC-4	90TC-4	SAMN03452288	
	F-6	-	SAMN02603935	Dairy product
	NCC2970	-	SAMN05510874	
4	DSM 20055	DSM20055	SAMN02797777	Sugar beet fermentation process
	RI-508	-	SAMN05717758	Cacao bean fermentation process
	779_LFER	-	SAMN03197989	Human respiratory tract
	222	-	SAMEA3158475	Cacao bean fermentation process

Supplementary Table 4. Description of the concentrations in $g.l^{-1}$ of the sugars and metabolites present in each of the media used in the microplate assays.

Medium	Glucose	Fructose	Sucrose	Ethanol	Acetic acid	Lactic acid
**Molasses**	1.668	1.608	15.284	0	32	101
**Fermented LAB A**	0	545	11.239	545	891	2.602
**Fermented LAB B**	0	500	12.04	422	886	2.687
**Fermented LAB C**	0	337	12.570	771	804	2.384
**Fermented A + Sugar**	1.668	1.762	18.484	541	884	2.584
**Fermented B + Sugar**	1.668	1.777	18.094	416	883	2.696
**Fermented C + Sugar**	1.668	1.884	17.929	763	797	2.377
**Molasses + Metabolites A**	1.668	1.608	15.284	557	883	2.338
**Molasses + Metabolites B**	1.668	1.608	15.284	475	847	2.392
**Molasses + Metabolites C**	1.668	1.608	15.284	871	808	2.144

Molasses refers to diluted molasses at a concentration of 20g l-1. Fermented refers to molasses fermented by lactic bacteria (LAB A,B and C). Fermented + sugar refers to bacteria fermented with the addition of sugars at a level equivalent to that found in the initial molasses. Molasses + metabolites refers to the initial molasses with the addition of metabolites at the level produced by bacteria during fermentation.

Supplementary Table 5. Description of the concentrations in g.l-1 of the sugars and metabolites present in each of the media used in the microplate assays.

Medium	Glucose	Fructose	Sucrose	Ethanol	Glycerol
Molasses	1.676	1.623	15.738	0	21
Fermented	1.208	3.482	5.814	4.634	813
Fermented + Sugar	1.270	3.329	15.157	4.445	801
Molasses + Metabolites	1.676	1.623	15.738	4.171	847

Molasses refers to diluted molasses at a concentration of 20g/l-1. Fermented refers to molasses fermented by Saccharomyces cerevisiae PE-2. Fermented + sugar refers to yeast fermented with the addition of sugars at a level equivalent to that found in the initial molasses. Molasses + metabolites refers to the initial molasses with the addition of metabolites (ethanol and glycerol) at the level produced by yeast during fermentation.
