# Supplementary material for: Strain dynamics of contaminating bacteria modulate the yield of ethanol biorefineries
Source: Nat Commun. 2024 Jun 22;15:5323. doi: 10.1038/s41467-024-49683-2 (PMC11193817; doi:10.1038/s41467-024-49683-2)
Supplement: Supplementary file 3 — Description of Additional Supplementary Files [file 41467_2024_49683_MOESM3_ESM.pdf]

Description of Additional Supplementary Files

Supplementary Data 1: Sampling timepoints and industrial metadata.

Description: Overview of industrial samples and their corresponding metadata.

Supplementary Data 2: Sequencing info and mapping stats of metagenomes.

Description: Overview of the sequencing quality and mapping stats of metagenomes from analysed industrial samples.

Supplementary Data 3: Metrics for genome and metagenome assemblies.

Description: Quality parameters from genome and metagenome assemblies.

Supplementary Data 4: MAGs taxonomy analysis and quality metrics.

Description: Metagenome Assembled Genomes (MAGs) from metagenomics samples, and subsequent taxonomic profiling from MAGs, showing *L. fermentum* and *L. amylovorus* as most abundant bacterial species for the majority of the industrial samples.

Supplementary Data 5: Sequences used for microbial community profiling

Description: Overview of sequences used for the microbial community profiling, including base length, identity to SILVA database sequence and SINA alignment score (in percentages).
