# Supplementary material for: Strain dynamics of contaminating bacteria modulate the yield of ethanol biorefineries
Source: Nat Commun. 2024 Jun 22;15:5323. doi: 10.1038/s41467-024-49683-2 (PMC11193817; doi:10.1038/s41467-024-49683-2)
Supplement: Supplementary file 9 — Reporting Summary [file 41467_2024_49683_MOESM9_ESM.pdf]

nature portfolio

Corresponding author(s): Morten Otto Alexander Sommer

Last updated by author(s): Apr 22, 2024

Reporting Summary

Nature Portfolio wishes to improve the reproducibility of the work that we publish. This form provides structure for consistency and transparency in reporting. For further information on Nature Portfolio policies, see our Editorial Policies and the Editorial Policy Checklist.

Statistics

For all statistical analyses, confirm that the following items are present in the figure legend, table legend, main text, or Methods section.

n/a	Confirmed	
☐	☒	The exact sample size (*n*) for each experimental group/condition, given as a discrete number and unit of measurement
☐	☒	A statement on whether measurements were taken from distinct samples or whether the same sample was measured repeatedly
☐	☒	The statistical test(s) used AND whether they are one- or two-sided<br>*Only common tests should be described solely by name; describe more complex techniques in the Methods section.*
☒	☐	A description of all covariates tested
☒	☐	A description of any assumptions or corrections, such as tests of normality and adjustment for multiple comparisons
☐	☒	A full description of the statistical parameters including central tendency (e.g. means) or other basic estimates (e.g. regression coefficient) AND variation (e.g. standard deviation) or associated estimates of uncertainty (e.g. confidence intervals)
☐	☒	For null hypothesis testing, the test statistic (e.g. *F*, *t*, *r*) with confidence intervals, effect sizes, degrees of freedom and *P* value noted<br>*Give P values as exact values whenever suitable.*
☒	☐	For Bayesian analysis, information on the choice of priors and Markov chain Monte Carlo settings
☒	☐	For hierarchical and complex designs, identification of the appropriate level for tests and full reporting of outcomes
☒	☐	Estimates of effect sizes (e.g. Cohen's *d*, Pearson's *r*), indicating how they were calculated

Our web collection on statistics for biologists contains articles on many of the points above.

Software and code

Policy information about availability of computer code

Data collection	Microsoft Excel Student 64 bits, Microsoft Word Student 64 bits, Benchling
Data analysis	Microsoft Excel Student 64 bits, SPAdes 3.12, IDBA_ud 1.1.1, MetaGeneMark 3.26, CD-HIT, CheckM Package 1.0.18, SINA 1.2.11, EZBioCloud 202201012, NGless 1.1, Kraken 0.10-5beta, Bracken 2.0, eggNOG 1.0.3-35-g63c274b, emapper 2.0, Prokka 1.14.0, ResFinder and antiSMASH, Genome Classifier tool, SequenceMatrix, MEGA X, R Studio, GraphPad Prism 8, FowJo 10.8.1

For manuscripts utilizing custom algorithms or software that are central to the research but not yet described in published literature, software must be made available to editors and reviewers. We strongly encourage code deposition in a community repository (e.g. GitHub). See the Nature Portfolio guidelines for submitting code & software for further information.

Data

Policy information about availability of data

All manuscripts must include a data availability statement. This statement should provide the following information, where applicable:

- Accession codes, unique identifiers, or web links for publicly available datasets
- A description of any restrictions on data availability
- For clinical datasets or third party data, please ensure that the statement adheres to our policy

All experimental data is available for sharing upon request. Metagenomic sequence data are deposited in the European Nucleotide Archive under accession number

PRJEB33675. Assembled genomes are under accession number PRJEB52385. The gene catalogue created in this study is available via the Zenodo data archive (DOI: 10.5281/zenodo.6807897).

Research involving human participants, their data, or biological material

Policy information about studies with human participants or human data. See also policy information about sex, gender (identity/presentation), and sexual orientation and race, ethnicity and racism.

Reporting on sex and gender	N/A
Reporting on race, ethnicity, or other socially relevant groupings	N/A
Population characteristics	N/A
Recruitment	N/A
Ethics oversight	N/A

Note that full information on the approval of the study protocol must also be provided in the manuscript.

Field-specific reporting

Please select the one below that is the best fit for your research. If you are not sure, read the appropriate sections before making your selection.

☒ Life sciences ☐ Behavioural & social sciences ☐ Ecological, evolutionary & environmental sciences

For a reference copy of the document with all sections, see nature.com/documents/nr-reporting-summary-flat.pdf

Life sciences study design

All studies must disclose on these points even when the disclosure is negative.

Sample size	Experiments were performed in triplicate in order to ensure a satisfactory sample size for further statistical analysis. This sample size was assessed experimentally, being the minimal sample size which allowed for statistically significant differences to be confirmed.
Data exclusions	No data was excluded from analysis.
Replication	All experiments were repeated at least twice to ensure reproducibility. We can confirm that all findings could be replicated.
Randomization	The industrial samples were analyzed and compared with other samples based on their industrial metadata (i.e. source within the industrial process). Laboratory experiments were performed taking the industrial context in order to design the competition and cross-feeding experiments between yeast and the specific bacterial species. We have analyzed solely the parameters that were relevant for the context of the study and, therefore, there was no need to control specific covariates.
Blinding	Collected samples from biorefineries received a unique code that was used during subsequent sequencing and bioinformatics analysis. The researchers performing such analyzes were unaware of the nature of the samples beforehand, in order to avoid any potential statistical bias. Both biorefineries also received codes during this project, anonymysing the source of the samples.

Reporting for specific materials, systems and methods

We require information from authors about some types of materials, experimental systems and methods used in many studies. Here, indicate whether each material, system or method listed is relevant to your study. If you are not sure if a list item applies to your research, read the appropriate section before selecting a response.

Materials & experimental systems

n/a	Involved in the study	
☒	☐	Antibodies
☒	☐	Eukaryotic cell lines
☒	☐	Palaeontology and archaeology
☒	☐	Animals and other organisms
☒	☐	Clinical data
☒	☐	Dual use research of concern
☒	☐	Plants

Methods

n/a	Involved in the study	
☒	☐	ChIP-seq
☐	☒	Flow cytometry
☒	☐	MRI-based neuroimaging

Plants

Seed stocks	N/A
Novel plant genotypes	N/A
Authentication	N/A

Flow Cytometry

Plots

Confirm that:

☒ The axis labels state the marker and fluorochrome used (e.g. CD4-FITC).

☒ The axis scales are clearly visible. Include numbers along axes only for bottom left plot of group (a 'group' is an analysis of identical markers).

☒ All plots are contour plots with outliers or pseudocolor plots.

☒ A numerical value for number of cells or percentage (with statistics) is provided.

Methodology

Sample preparation	A sample from each well (10µl) was taken after the overnight cultivation, and was transferred to a new microplate and diluted in 190µl PBS buffer (pH 7.4). Yeast and bacteria populations were resolved via front and side scatter comparison (SSC versus FSC).
Instrument	BD LSRFortessa™, BD Biosciences, Franklin Lakes, New Jersey, USA.
Software	FlowJo 10.8.1
Cell population abundance	Population analyzes were performed in samples obtained from controlled experiments. In that case, yeast and bacteria populations were well defined and without any external contaminant. In the end of the experiments the ration between yeast and bacteria populations was ca. 100:1.
Gating strategy	Doublet were discriminated by applying a gating strategy such as FSC-H x FSH-A. In the singlet gating, yeast and bacteria populations were resolved via front and side scatter comparison (FSC x SSC).

☒ Tick this box to confirm that a figure exemplifying the gating strategy is provided in the Supplementary Information.
