# Supplementary material for: Strain dynamics of contaminating bacteria modulate the yield of ethanol biorefineries
Source: Nat Commun. 2024 Jun 22;15:5323. doi: 10.1038/s41467-024-49683-2 (PMC11193817; doi:10.1038/s41467-024-49683-2)
Supplement: Supplementary file 2 — Peer Review File [file 41467_2024_49683_MOESM2_ESM.pdf]

nature portfolio

Peer Review File

Strain dynamics of contaminating bacteria modulate the yield of ethanol biorefineries

Open Access This file is licensed under a Creative Commons Attribution 4.0 International License, which permits use, sharing, adaptation, distribution and reproduction in any medium or format, as long as you give appropriate credit to the original author(s) and the source, provide a link to the Creative Commons license, and indicate if changes were made. In the cases where the authors are anonymous, such as is the case for the reports of anonymous peer reviewers, author attribution should be to 'Anonymous Referee' followed by a clear attribution to the source work. The images or other third party material in this file are included in the article's Creative Commons license, unless indicated otherwise in a credit line to the material. If material is not included in the article's Creative Commons license and your intended use is not permitted by statutory regulation or exceeds the permitted use, you will need to obtain permission directly from the copyright holder. To view a copy of this license, visit http://creativecommons.org/licenses/by/4.0/.

Reviewers' Comments:

Reviewer #1:
Remarks to the Author:
Interestingly, all referees voiced the same concerns about incremental novelty of this work, which leaves the question open if Nature Communications is the most appropriate outlet. But as I pointed out before, this is in the end an editorial decision.
The authors have thoroughly revised their manuscript and I think it is ready for publication.

Reviewer #2:
Remarks to the Author:
The manuscript has been improved significantly and most points addressed.

I believe Figure 1D is a cluster of nanofibers rather than single fibers - similar to Small Burns 2021 or ACS nano Zhang et al. 2023 publications. Potentially the background objects in the TIRF microscope image are single fibers which have bundled up.

Reviewer #3:
Remarks to the Author:
The authors have made significant revisions of the manuscript in light of my review (as well as the other reviews). All three reviewers discussed novelty with the authors much more clearly pointing out what is new in the current manuscript. If the editors are fine with the novelty discussion, I am pleased with the effort of the authors in revising the manuscript significantly in light of the reviews.

I gave reference 5 in the response letter a look with respect to Mg+2 effects on DNA and DNA origami structures. This led me down a bit of a mono- vs di- vs Reviewers' Comments:

Reviewer #1:
Remarks to the Author:
The manuscript describes a survey of ethanol production facilities in Brazil that examines the bacterial consort of ethanol producing yeast strains and links changes in performance to different Lactobacillus species. This report adds valuable information to understanding the controls on ethanol production, however I was surprised at the tools used to analyze the metagenomes and lack of any gene expression and/or proteomic data to support the conclusions in the manuscript:

1) Many of the conclusions in the manuscript are based on gene abundances in the metagenomes rather than gene expression or protein profiles. I think that these data would add a great deal to the understanding of metabolite flows and the mechanisms of beneficial or inhibitory bacteria.

2) The conclusions are based on gene-centric metagenomics, based on the size of the co-assembly (254MB) it should be possible to use modern binning tools to reconstruct MAGs from the assembly and describe the metabolic potential in a genome-centric way. This analysis may also provide information on strain variation, which the authors have shown in their cultivation based methods to be distinguishing in performance.

3) The use of SSU rRNA genes obtained directly from the assembled metagenomes for relative quantification is problematic, as the SSU rRNA genes are usually not well assembled and it is difficult to get consistency in the size of the individual rRNA gene sizes. Why did the authors not use ribosomal proteins genes, which are better assembled and can give more phylogenetic information. In fact having the MAGs would give much more information about the identity of the microbiomes.

I think the more comprehensive approach that includes gene epxression/proteins and MAGs will give a more complete picture and provide more confidence that the culture based studies reflect what is occurring in the microbiomes.

Reviewer #2:
Remarks to the Author:
The manuscript by Oliveira Lino et al. reports findings from metagenomic sampling of ethanol biorefineries. They identify species that are correlated with ethanol yield differences. Certain strains of L. fermentum appear to be associated with lower ethanol yields. The authors also demonstrate in vitro scenarios where temperature can modulate growth of detrimental L. fermentum strains.

While the topic is of high biotechnological importance, the study is very much descriptive in its current stage. The abstract refers to "complex ecological interactions", though neither demonstrated nor discussed. I have following suggestions for authors' consideration:

1. The yeast-bacteria interaction are often bi-directional (e.g., see review by Watanabe et al. FEMS Yest Res 2019 for bacteria-yeast; and Ponomarova et al. Cell Systems 2017 for yeast-bacteria interaction; there are more studies that can be traced from these references). It will be insightful to map (at least phenotypically, ideally also using some molecular readouts) interactions between yeast and bacterial strains from the current study; and to discuss the implications for ethanol yield and community dynamics.

2. Any metadata on, e.g., temperature and yield, from additional biorefineries will be useful as additional support.

3. Methodological clarity on High and low batch performances: How are these calculated? What is the actual difference in yields here? It's not clear from the data shown whether the differences in community abundances are a problem or not.

4. Line 128 – 130: "Surprisingly, high-performing batches were not characterised by a dominance in eukaryotes; low-performing batches were not necessarily dominated by bacteria.": Total biomass of eukaryotes be more important here rather than the proportional abundance.

Other comments
1. Figure 1C: y-axis title is unclear.

2. Figure 1D: From which stage are samples taken for this figure?

3. Figure 3B: How were these associations calculated? What do the circles represent?

4. Line 431 - Industrial performance calculation:
1. Is this used to define the low/high batch performances? Unclear where this equation fits in with the results. Furthermore, how is the cut-off between low and high defined?
2. Why is the industrial performance not just the ethanol yield directly? Please explain the significance of the other parameters as not all readers will be familiar with these.

Reviewer #3:
Remarks to the Author:

Lino et al. reported the industrial bioethanol microbiome profiles across all processing stages based on shotgun metagenomics sequencing on 56 samples from different industrial steps. They explored 7 Lactic acid bacteria, such as L. amylovorus and L. fermentum, were dominant in the industrial bioethanol microbiome. They also found an isolated L. fermentum strain C was associated with lowered production and reduced ethanol yield by ~5%. Another interesting observation was temperatures could be used to control the abundance of L. fermentum strains during fermentation. I am not an industrial microbiologist, so I only comment on metagenomic data analysis.

Major comments:
1. I think the metagenomic sequencing data are good resource to decipher microbial compositions using metagenome-assembled genomes (MAGs). MAGs can also help to explore genomic variants and microbial composition diversity between samples (PMID: 30661755, PMID: 32690973, PMID: 30745586). It may help uncover more potential strains correlated with bioethanol fermentation. Besides the gene catalog, I highly suggest the authors to construct a catalog of reference genomes.
2. It has proved unreliable to annotate ribosomal RNA genes from metagenomic assembly (PMID: 30745586). I doubt the accuracy of taxonomic profiling using ribosomal RNA genes in this study. The authors could update the profiling strategy using MAGs or marker genes.

3. This study discovered the strains of L. fermentum led to different ethanol yields, but little is done to clarify the reasons in terms of their genomic characteristics. What are the genomic variations associated with the different metabolites they produce? Are they sensitive/resistant to any specific antibiotics?

Minor comments:
1) Page 4, line 69: “effected”, typo ?
2) Page 7, line 123: the relative abundances of eukaryotic and bacterial populations should be shown in separate tables. “Supplementary Tables 1 and 2” should be bolded.
3) Page 8, line 141: “Fig.1B”, typo? As no LPS and PTD information is shown in Fig. 1B.
4) L.amylovorus and L.fermertum showed opposing effects. What are their potential functional differences? What are their contributions to the KEGG modules? These can be calculated by the methods described in PMID: 30531976.
5) Page 14, line 241: More details are needed for the novel discovered strains, such as G. stearothermophilus. How about its Spearman's rank correlation coefficient with bioethanol?
6) Page 14, Figure 3: Could you provide the direct associations between L. fermentum’s abundance and bioethanol production?
7) The analyses presented in Figure 4 are not sufficient. I would like to know more about their genomic differences?
8) Page 27: line 494, NCBI ref genome should be given.
9) How did you merge the assemblies from SPAdes and idba-hybrid to calculate the metrics in Supplementary Table 4?
10) Why was idba_ud used for metagenome co-assembly? metaSPAdes and megahit are the better choices. I wonder if these advanced assembler could improve contig N50, as the current N50 is only 1.2k.
11) Page 29: line 532, please provide the full name of SSU.rivalent/multivalent cation rabbit hole with respect to effects on DNA (in comparison with or in conjunction with DNA condensing with spermine/spermidine/histones). Since I am not immersed in the DNA origami field, I may simply be using language a bit differently than what is invoked when discussing DNA in particular. My only point about the Mg+2 is that it is likely condensed/complexed with the DNA as opposed to loosely associated with it like a monovalent ion would be. I was thinking the condensation (whether there is water also present or not) could help lead to conformational changes in the DNA instead of just complexation of opposite charges. Looking at the literature it seems Mg+2, while complexing with DNA, is not sufficient to cause collapse/assembly of the DNA (as opposed to what is observed with trivalent cations/multiamines/histones). Perhaps a semantic point is that in the general polymer physics community, one wouldn't call that "screening" but rather would describe it as a complexation

with screening occurring with soluble, loosely associated ions (no matter the valency). But, the important point is that Mg+2 helps stabilize origami by complexing out charge of the DNA chain (as opposed to screening it while in solution). Not important here-I think the manuscript is solid and is worthy of publication.

Reviewers' rebuttals

Reviewer #1 (Remarks to the Author):

The manuscript describes a survey of ethanol production facilities in Brazil that examines the bacterial consort of ethanol producing yeast strains and links changes in performance to different Lactobacillus species. This report adds valuable information to understanding the controls on ethanol production, however I was surprised at the tools used to analyze the metagenomes and lack of any gene expression and/or proteomic data to support the conclusions in the manuscript:

1) Many of the conclusions in the manuscript are based on gene abundances in the metagenomes rather than gene expression or protein profiles. I think that these data would add a great deal to the understanding of metabolite flows and the mechanisms of beneficial or inhibitory bacteria.

Reply: Thank you for your comment. Indeed, gene expression analysis could aid in the understanding of the interplay between yeast and bacteria. Unfortunately, it is outside the scope of this study as the samples would have to be prepared differently at collection for metatranscriptomic and metaproteomic analysis and we do not have any samples available for such analysis at this point in time.

2) The conclusions are based on gene-centric metagenomics, based on the size of the co-assembly (254MB) it should be possible to use modern binning tools to reconstruct MAGs from the assembly and describe the metabolic potential in a genome-centric way. This analysis may also provide

information on strain variation, which the authors have shown in their cultivation based methods to be distinguishing in performance.

Reply: Thank you for the great suggestion. We have now implemented a new MAG pipeline that integrates modern binning tools. See Methods section.

"**Metagenome-assembled genomes (MAG) pipeline**

In this section, we present the comprehensive and detailed methodology for the analysis of metagenomic raw data, addressing every aspect from initial quality control to the final annotation and classification stages/MAG production:

1. Quality Control and Trimming: The process starts with paired-end reads of 150 bp. FastQc (v0.12.1) inspects the raw reads for quality control, providing a preliminary assessment of potential issues in the sequence data. Subsequent trimming is performed using bbduk (v39.00), a kmer-based decontamination tool. This step involves the removal of adapter sequences and the exclusion of sequences with a Phred score less than 33 and a minimum read length of 100 bp. The parameters set for this process include qin=33 for input quality offset, hdist=1 for Hamming distance in error correction, and trimq=30 for quality trimming, among others.

2. Assembly and Dereplication: The initial co-assembly of the trimmed reads is executed with MEGAHIT (v1.2.9), which assembles by incrementing odd-number kmers to avoid palindromes. The subsequent dereplication step involves CD-HIT-EST (v4.8.1), where contigs with a minimum of 99% global sequence similarity are combined to eliminate redundant sequences. A secondary assembly is then performed with CAP3, focusing on contigs with an identity and overlap length cutoff of ≥ 97%.

3. Contig Quality Control: After assembly, the contigs undergo quality control using Quast.

4. Coverage Analysis and Binning: The sequencing depth or coverage is determined by aligning the short reads to the co-assembled contigs using BWA MEM (v0.7.17-r1188) and further processed with Samtools (v1.16.1) and Bedtools (v2.31.0). This data is then utilized in the binning process, employing state-of-the-art tools like MetaBAT2, MaxBin2, and CONCOCT. These tools are chosen to minimize biases and maximize accuracy, with each providing a unique approach to binning. The outputs from these tools are aggregated using DASTool (v1.1.6), which refines the bins by dereplicating contigs and selecting high-quality candidates.

5. Quality Check of Binned Contigs: The quality of the binned contigs is assessed using CheckM (v1.2.2) with its "lineage wf" workflow. This step is crucial for evaluating the completeness and contamination levels of the binned contigs. Meta-QUAST is also employed for additional quality control."

We have now included analysis for strain-specific functional characteristics for each *Lactobacillus fermentum* strain in Supp. Fig. 7 using the MAGs produced.

3) The use of SSU rRNA genes obtained directly from the assembled metagenomes for relative quantification is problematic, as the SSU rRNA genes are usually not well assembled and it is difficult to get consistency in the size of the individual rRNA gene sizes. Why did the authors not use ribosomal proteins genes, which are better assembled and can give more phylogenetic information. In fact having the MAGs would give much more information about the identity of the

microbiomes. I think the more comprehensive approach that includes gene expression/proteins and MAGs will give a more complete picture and provide more confidence that the culture based studies reflect what is occurring in the microbiomes.

Reply: Thank you for the great suggestion. We now present the taxonomy profiling from MAGs using state-of-the-art methods. See Methods section.

"Functional Annotation and Taxonomical Identification of MAGs

For functional annotation, tools like CAT/BAT, PROKKA, and eggNOG are used, with Prodigal (v2.6.3) serving as the protein predictor. The predicted proteins are compared against the NCBI nonredundant database using DIAMOND (v2.1.8.162). Taxonomical identification is performed using KRAKEN2 (v2.1.3), a kmer-based method for fast and accurate classification, using its standard database."

The relative abundances across all samples are presented in Supp. Fig 7A.

Reviewer #2 (Remarks to the Author):

The manuscript by Oliveira Lino et al. reports findings from metagenomic sampling of ethanol biorefineries. They identify species that are correlated with ethanol yield differences. Certain strains of *L. fermentum* appear to be associated with lower ethanol yields. The authors also demonstrate in vitro scenarios where temperature can modulate growth of detrimental *L. fermentum* strains. While the topic is of high biotechnological importance, the study is very much descriptive in its current stage. The abstract refers to "complex ecological interactions", though neither demonstrated nor discussed. I have following suggestions for authors' consideration:

1) The yeast-bacteria interaction are often bi-directional (e.g., see review by Watanabe et al. FEMS Yest Res 2019 for bacteria-yeast; and Ponomarova et al. Cell Systems 2017 for yeast-bacteria interaction; there are more studies that can be traced from these references). It will be insightful to map (at least phenotypically, ideally also using some molecular readouts) interactions between yeast and bacterial strains from the current study; and to discuss the implications for ethanol yield and community dynamics.

Reply: Thank you for your suggestion. In order to address such interactions (from a phenotypic perspective), we ran additional cultivations in which we compared the growth profiles (final OD and specific growth rate; μ) of the yeast *S. cerevisiae* PE-2 inoculated in 1) diluted sugarcane molasses (20 g.l-1 TRS) against molasses previously fermented with each of the 3 *L. fermentum* strains (A, B and C) 2) with or 3) without the supplementation of sugars (to restore the original sugar titres) and, 4) with fresh molasses spiked with key bacterial metabolites (i.e. acetic and lactic acids). Based on the observed results, it seems that the inhibition of yeast growth is not solely driven by organic acid production by the bacteria, but probably by the lack of essential nutrients in the media that was previously cultivated with bacteria, suggesting competition for nutrients among them.

Considering the bidirectionality of microbial interactions, we also evaluated the impact of yeast metabolism on the growth of the *L. fermentum* isolates. For that, the cultivation of the isolates in 1) freshly diluted molasses (20 g.l-1 TRS) was compared against 2) depleted molasses from yeast cultivation; 3) depleted molasses from yeast cultivation supplemented with a mixture of sucrose, glucose and fructose (to restore the original sugar titres); and 4) molasses spiked with yeast metabolites (i.e. ethanol and glycerol). The results suggested that one of the strains (strain C) might be fitter for faster growth during the fermentation, when compared to other *L. fermentum* strains,

and environmental conditions might play a big role in defining which dominant strain would overtake the *L. fermentum* population in the process.

All these observations were added in the revised version of the manuscript, within the section "Metabolic differences drive strain-specific impact on fermentation performance" and as Supplementary Material.

2) Any metadata on, e.g., temperature and yield, from additional biorefineries will be useful as additional support.

Reply: Thank you for your comment. We do agree that it would be valuable and interesting to present additional performance parameters from other biorefineries. But such datasets were not available for this study. They are commonly treated as confidential information within these companies and were only provided because we had an open research collaboration ongoing, and only after we ensured that they would not be published with any information correlating it to the specific biorefinery.

3) Methodological clarity on High and low batch performances: How are these calculated? What is the actual difference in yields here? It's not clear from the data shown whether the differences in community abundances are a problem or not.

Reply: Thank you for your question. High and low batch performances were calculated using the equation described in the methods section entitled "Industrial performance calculation". The definition of this new parameter was partly necessary in order to normalize and directly compare

the yields of these 2 biorefineries. As mentioned in the manuscript, several parameters (some from operational nature) affect the yield calculation. Therefore, the yield values varied significantly between both mills. Also, in order to better capture any potential influence of microbial community dynamics in the process performance we have decided to incorporate other relevant parameters in this performance metric, such as yeast viability, microbial cell counts and acidity – this parameter is later demonstrated to have a strong correlation with yield when analyzing the weekly averages yearly data of the biorefineries (Figure 3A). These average values are less sensitive to daily fluctuations in process and, therefore, more stable to work with.

The yield values from both mills from the sampling dates are as following:

Mill A High – 89.79%

Mill A Medium – 88.36%

Mill A Low – 79.71%

Mill B High – 96.1%

Mill B Medium – mill was halted, weekly average was 86.7%

Mill B Low – 86.4%

4) Line 128 – 130: "Surprisingly, high-performing batches were not characterised by a dominance in eukaryotes; low-performing batches were not necessarily dominated by bacteria.": Total biomass of eukaryotes be more important here rather than the proportional abundance.

Reply: Thank you for your comment. In the Brazilian bioethanol production process, yeast growth is quite limited. Usually, yeast growth during fermentation is kept below 10%, and yeast biomass

is kept within similar levels throughout the crop season[1]. Moreover, it is difficult to infer the total eukaryote biomass from the metagenomic dataset due to contamination from plant material, naturally present in the samples and the selected sample processing strategy.

Other comments

1) Figure 1C: y-axis title is unclear.

Reply: Thank you for your observation. We have included a further explanation in the figure description as “The y-axis indicates the fold change in the eukaryote to bacteria ratio. Higher fold values indicate a greater eukaryote abundance, and lower fold values a higher prokaryotic abundance”.

2) Figure 1D: From which stage are samples taken for this figure?

Reply: The samples were obtained from the following unitary processes: Acid wash, fermentation (beginning, middle and end of fermentation) and after centrifugation.

3) Figure 3B: How were these associations calculated? What do the circles represent?

Reply: Thank you for your question. As described in the Methods section “Statistical Analyses”, the associations were calculated via Spearman’s correlation coefficient, which was calculated for each pair of industrial metadata variables, and between metadata variables and species abundances.

False discovery rate (FDR) was calculated using Benjamini-Hochberg (BH) method, with FDR < 0.1 used as cut-off. In this case, the relative abundance of each microbial species from industrial samples was compared against industrial process metadata from these given samples. Circles represent significant correlations between given parameters. The bigger the circle, higher is the correlation (Spearman's rho value). **A description of this correlation has been added in the figure's subtitle.**

4) Line 431 - Industrial performance calculation:

1. Is this used to define the low/high batch performances? Unclear where this equation fits in with the results. Furthermore, how is the cut-off between low and high defined?

Reply: Thank you for your question. Indeed, this calculation is used to define the low/high batch performance values. We have added a sentence in the Results section "Lactic acid bacteria dominate the contaminant microbial community and their interplay influences process performance" about this equation and how it's linked with the definition of low and high batch performances. The definition of low and high batch performance was comparative, based on the highest and lowest yield obtained during samplings. Furthermore, we also compared the low and high yield samples from the annual yield average of the particular mill, ensuring we have indeed sampled extreme values.

2) Why is the industrial performance not just the ethanol yield directly? Please explain the significance of the other parameters as not all readers will be familiar with these.

Reply: Thank you for your question. As discussed before, ethanol yield calculation differed significantly between both biorefineries. After further investigation we figured out that both mills were using different methods to calculate this parameter (i.e. mass balance on mill A and via co-products on mill B, personal communication). Moreover, ethanol yield is a parameter that shows very inconsistent trends in short periods of time[2]. Also, process related issues, like industry downtime in the day before (due to rain, or lack of steam, for example) might artificially reduce the yield in one day and overestimate it on the second, due to the accumulated fermented beer that was not processed in a given operation turn. In order to use yield as the sole process parameter, one would have to perform a much longer and thorough sampling campaign, which would be impossible based on the analytical design of this study. Moreover, our intention was to correlate this particular process parameter with other relevant parameters which are more stable and directly measurable. We have not only identified extremely significant correlations between acidity and yield, when using a year-long log of the process performance, but also acidity with microbial community composition. These results show that this parameter is actually an extremely useful and low-cost analysis data to follow, in order to indirectly assess the contaminant community composition and anticipate its potential impacts on the process, potentially changing the way these industries respond to contamination. This was only possible when taking this multi-parameter approach.

Reviewer #3 (Remarks to the Author):

Lino et al. reported the industrial bioethanol microbiome profiles across all processing stages based on shotgun metagenomics sequencing on 56 samples from different industrial steps. They explored 7 Lactic acid bacteria, such as *L. amylovorus* and *L. fermentum*, were dominant in the industrial

bioethanol microbiome. They also found an isolated L. fermentum strain C was associated with lowered production and reduced ethanol yield by ~5%. Another interesting observation was temperatures could be used to control the abundance of *L. fermentum* strains during fermentation. I am not an industrial microbiologist, so I only comment on metagenomic data analysis.

Major comments:

1) I think the metagenomic sequencing data are a good resource to decipher microbial compositions using metagenome-assembled genomes (MAGs). MAGs can also help to explore genomic variants and microbial composition diversity between samples (PMID: 30661755, PMID: 32690973, PMID: 30745586). It may help uncover more potential strains correlated with bioethanol fermentation. Besides the gene catalog, I highly suggest the authors to construct a catalog of reference genomes.

Reply: Thank you for the great suggestion. We have now implemented a novel MAG pipeline using state-of-the-art tools. This produces a catalog of reference genomes that are now used for subsequent downstream analyses.

We now present the comprehensive and detailed methodology for the analysis of metagenomic raw data, addressing every aspect from initial quality control to the final annotation and classification stages/MAG production:

1. Quality Control and Trimming: The process starts with paired-end reads of 150 bp. FastQc (v0.12.1) inspects the raw reads for quality control, providing a preliminary assessment of potential issues in the sequence data. Subsequent trimming is performed using bbduk (v39.00), a

kmer-based decontamination tool. This step involves the removal of adapter sequences and the exclusion of sequences with a Phred score less than 33 and a minimum read length of 100 bp. The parameters set for this process include qin=33 for input quality offset, hdist=1 for Hamming distance in error correction, and trimq=30 for quality trimming, among others.

2. Assembly and Dereplication: The initial co-assembly of the trimmed reads is executed with MEGAHIT (v1.2.9), which assembles by incrementing odd-number kmers to avoid palindromes. The subsequent dereplication step involves CD-HIT-EST (v4.8.1), where contigs with a minimum of 99% global sequence similarity are combined to eliminate redundant sequences. A secondary assembly is then performed with CAP3, focusing on contigs with an identity and overlap length cutoff of ≥ 97%.

3. Contig Quality Control: After assembly, the contigs undergo quality control using Quast.

4. Coverage Analysis and Binning: The sequencing depth or coverage is determined by aligning the short reads to the co-assembled contigs using BWA MEM (v0.7.17-r1188) and further processed with Samtools (v1.16.1) and Bedtools (v2.31.0). This data is then utilized in the binning process, employing state-of-the-art tools like MetaBAT2, MaxBin2, and CONCOCT. These tools are chosen to minimize biases and maximize accuracy, with each providing a unique approach to binning. The outputs from these tools are aggregated using DASTool (v1.1.6), which refines the bins by dereplicating contigs and selecting high-quality candidates.

5. Quality Check of Binned Contigs: The quality of the binned contigs is assessed using CheckM (v1.2.2) with its "lineage wf" workflow. This step is crucial for evaluating the completeness and contamination levels of the binned contigs. Meta-QUAST is also employed for additional quality control.

2) It has proved unreliable to annotate ribosomal RNA genes from metagenomic assembly (PMID: 30745586). I doubt the accuracy of taxonomic profiling using ribosomal RNA genes in this study. The authors could update the profiling strategy using MAGs or marker genes.

Reply: Thank you for the great suggestion. We now present the taxonomy profiling from MAGs using state-of-the-art methods. See Methods section.

"Functional Annotation and Taxonomical Identification of MAGs

For functional annotation, tools like CAT/BAT, PROKKA, and eggNOG are used, with Prodigal (v2.6.3) serving as the protein predictor. The predicted proteins are compared against the NCBI nonredundant database using DIAMOND (v2.1.8.162). Taxonomical identification is performed using KRAKEN2 (v2.1.3), a kmer-based method for fast and accurate classification, using its standard database."

The relative abundances across all samples are presented in Fig 6A.

3) This study discovered the strains of *L. fermentum* led to different ethanol yields, but little is done to clarify the reasons in terms of their genomic characteristics. What are the genomic

variations associated with the different metabolites they produce? Are they sensitive/resistant to any specific antibiotics?

Reply: We have now added a plot on Supp Fig 7B for strain-specific metabolic characterization. Moreover, at least one of the mills did not apply antibiotics in the year of sampling, as they avoid using those since it has a negative economic impact in their operations (i.e. they are not able to sell their surplus yeast for feed applications for at least one week).

Minor comments:

1) Page 4, line 69: “effected”, typo ?

Reply: Thank you for your observation. This word was corrected for “affected”.

2) Page 7, line 123: the relative abundances of eukaryotic and bacterial populations should be shown in separate tables. “Supplementary Tables 1 and 2” should be bolded.

Reply: Thank you for your comment. The abundance of both populations are linked from actual industrial metadata provided by the mills and, therefore, were kept in the same table, together with other relevant industrial parameters. We appreciate your suggestion, but we believe keeping these data altogether in this table makes it easier for comparing relevant process parameters. We have bolded the Supplementary Tables 1 and 2 sentence.

3) Page 8, line 141: “Fig.1B”, typo? As no LPS and PTD information is shown in Fig. 1B.

Reply: Thank you for your comment. Both LPS (Glycan Metabolism) and PTD (Membrane Transport) are shown in the figure, as the bottom right 2 blocks.

4) *L. amylovorus* and *L. fermertum* showed opposing effects. What are their potential functional differences? What are their contributions to the KEGG modules? These can be calculated by the methods described in PMID: 30531976.

Reply: Thank you for your question. The core difference between *L. amylovorus* and *L. fermentum* lies in their central carbon metabolism. While *L. fermentum* shows an exclusive heterofermetative metabolism, *L. amylovorus* shows a facultative heterofermentative. Under the fermentation conditions, *L. amylovorus* solely produces lactic acid, whereas *L. fermentum* produces a mix of acetic and lactic acid, which is significantly more detrimental to yeast metabolism[3]. Also, *L. amylovorus* is able to generate a pool of acetaldehyde, which *S. cerevisae* can utilize as an electron acceptor, reducing glycerol formation and increasing ethanol and biomass production[4].

5) Page 14, line 241: More details are needed for the novel discovered strains, such as *G. stearothermophilus*. How about its Spearman's rank correlation coefficient with bioethanol?

Reply: Thank you for your question. *G. stearothermophilus* has a weak correlation with bioethanol (i.e. spearman rank of around 0.25 for daily ethanol yields). Moreover, its low abundance in the microbial population (i.e. less than 1%) raises questions if its presence is relevant to any process parameter. However, its presence is indeed new, and deserves further investigations.

6) Page 14, Figure 3: Could you provide the direct associations between *L. fermentum*'s abundance and bioethanol production?

Reply: Thank you for your question. There is no real direct association between *L. fermentum*'s abundance and bioethanol production. The reasons for this, as we further explain in the manuscript are: 1) ethanol yield measurements are unreliable when used as a sole performance indicator, since it is directly correlated with operational (idle days due to rains, lower feedstock input, low steam production) and fermentation (contaminant community composition, acidity levels, feedstock quality and presence of environmental contaminants, etc.) issues and; 2) as we also demonstrate in the manuscript, is not only the *L. fermentum* abundance that matters, but rather its ratio between *L. amylovorus* (a beneficial strain for ethanol production) and its strain level diversity.

7) The analyses presented in Figure 4 are not sufficient. I would like to know more about their genomic differences?

Reply: Thank you for your question. We have now performed a Metagenome-Assembled Genomes Analysis (MAGs) and included the observed differences from these 3 strains (**Figure 6**).

8) Page 27: line 494, NCBI ref genome should be given.

Reply: Thank you for pointing this out. This list would be considerably extensive, due to the number of identified species. Since NCBI provides one specific reference genome per identified species, we believe it is best to simply refer to NCBI.

9) How did you merge the assemblies from SPAdes and idba-hybrid to calculate the metrics in Supplementary Table 4?

Reply: Thank you for your question. Two modifications were made in the source code before compiling IDBA_UD: (1) in file src/basic/kmer.h, kNumUint64 was changed from 4 to 8 to allow maximum kmer length beyond 124; (2) in file src/sequence/short_sequence.h, kMaxShortSequence was set to 512 to support longer read length.

10) Why was idba_ud used for metagenome co-assembly? metaSPAdes and megahit are the better choices. I wonder if these advanced assembler could improve contig N50, as the current N50 is only 1.2k.

Thank you for the great suggestion. We have now used megahit in the pipeline. The current N50 is > 14 k.

11) Page 29: line 532, please provide the full name of SSU.

Reply: Thank you for your comment. In the manuscript we discriminate between the different subunits used for both bacteria and yeast taxonomy (16S and 18S, respectively).

References

1. Basso, L. C., Basso, T. O. & Rocha, S. N. Ethanol Production in Brazil : The Industrial Process and Its Impact on Yeast Fermentation. *Biofuel production recent developments and prospects* **1530**, 85–100 (2011).

2. Bermejo, P. M. *et al.* Ethanol yield calculations in biorefineries. *FEMS Yeast Research* **21**, foab065 (2021).

3. Basso, T. O.; Gomes, F. S.; Lopes, M. L.; Amorim, H. V.; Eggleston, G.; Basso, L. C. Homo- and heterofermentative lactobacilli differently affect sugarcane-based fuel ethanol fermentation. *Anthonie Van Leeuwenhoek* **105** (1), 169 - 177 (2014).

4. Lino, F. S. O.; Bajic, D.; Villa, J. C. C.; Sánchez, A.; Sommer, M. O. A. Complex yeast-bacteria interactions affect the yield of industrial ethanol fermentation. *Nature Communications* **12**, 1498 (2021)

Reviewers' Comments:

Reviewer #1:
Remarks to the Author:
The authors have addressed my concerns and the manuscript is ready for publication.

Reviewer #2:
Remarks to the Author:
I am generally satisfied with the authors' response. There are a few important points that I recommend to address:

1. "complex ecological interactions" is not justified based on the data provided which rather suggests nutrient competition and possibly some cross-feeding between two members. I therefore recommend to remove "complex"

2. Since there are possible confounders for which there is no data available due to industrial confidentiality, it will be important to explicitly discuss this.

3. Yeast vs bacterial biomass calculation can easily be done using known cell masses.

Reviewer #3:
Remarks to the Author:
I am happy to see the revised manuscript has included more genomic analysis, especially on MAGs, but I still have several questions about the analysis.
1. I am curious how the co-assembly pipeline by Megahit works. The project produced 2.5*10^5 Gb data from 56 samples. I believe the co-assembly (merge all sequencing data) would require ~1PB RAM based on the comments from MEGAHIT's author https://github.com/voutcn/megahit/issues/144
2. The binning tools adopted are not state-of-the-art. I suggest trying VAMB, Semibin2, COMEBin et al, which could significantly increase the number of near complete bins and strain-resolved bins.
3. Kraken2 is not a tool designed for MAG annotation, as it can only annotate single sequences, e.g. contigs. I'm curious how it was utilized to annotate MAGs.
4. There seems to be a lack of information on co-assembly and the quality of MAGs, such as completeness and contamination, as well as the number of low-quality, medium-quality, and high-quality bins.
5. I couldn't find any details on how the authors identified L. amylovorus and L. fermentum from the MAGs. Are there two strain-resolved MAGs representing these two strains? What are their completeness and contamination? It's generally quite challenging to generate strain-resolved MAGs using short-read assembly and binning.

Reviewers rebuttal 2nd review

Reviewer #1 (Remarks to the Author):

The authors have addressed my concerns and the manuscript is ready for publication.

Reviewer #2 (Remarks to the Author):

I am generally satisfied with the authors' response. There are a few important points that I recommend to address:

1. "complex ecological interactions" is not justified based on the data provided which rather suggests nutrient competition and possibly some cross-feeding between two members. I therefore recommend to remove "complex"
Reply: Thank you for your comment. We have removed the term from the manuscript.

2. Since there are possible confounders for which there is no data available due to industrial confidentiality, it will be important to explicitly discuss this.
Reply: Thank you for your comment. We have included the disclaimer text "Important to mention that, due to confidentiality reasons, not all process data has been provided, leading to possible confounders without proper data available" on line 109 of the revised manuscript.

3. Yeast vs bacterial biomass calculation can easily be done using known cell masses.
Reply: We appreciate the reviewer's suggestion. Unfortunately it is not possible for us to identify the individual cell masses from each species present in the complex community found in the fermentation process. It could also be misleading to use known masses obtained from pure cultures, since the physiological conditions of these cultures would be significantly different from the one found in the actual fermentation process. The competition for nutrients and energy, the inhibitory metabolism of different species, and perhaps even quorum sense mechanisms can all cause changes in cell volumes and dimensions, making a direct comparison imprecise[1,2].

Reviewer #3 (Remarks to the Author):

I am happy to see the revised manuscript has included more genomic analysis, especially on MAGs, but I still have several questions about the analysis.
1. I am curious how the co-assembly pipeline by Megahit works. The project produced 2.5*10^5 Gb data from 56 samples. I believe the co-assembly (merge all sequencing data) would require ~1PB RAM based on the comments from MEGAHIT's author https://github.com/voutcn/megahit/issues/144
Reply: We acknowledge and concur that a co-assembly of data from all 56 samples using Megahit would demand extensive computational resources. It's important to note that we opted for a per-sample assembly approach with Megahit to ensure specificity. This method was chosen not only to manage computational demands but also to accurately reflect the diversity inherent in different fermentation processes and mills. Post-assembly, we implemented quality control measures such as dereplication to remove any redundant sequences, enhancing the integrity of our assemblies.

Moreover, by opting to assemble each sample individually, we were effectively able to screen out and discard potential chimeric reads, thereby minimizing the occurrence of misassembled contigs. This approach has allowed us to produce more precise and cleaner assemblies, with a specific focus on capturing the genetic material relevant to each sample's unique microbial composition.

2. The binning tools adopted are not state-of-the-art. I suggest trying VAMB, Semibin2, COMEBin et al, which could significantly increase the number of near complete bins and strain-resolved bins.
Reply: Thank you for your valuable suggestion. Following your advice, we conducted a binning experiment with Vamb, and the comprehensive results of the binning quality have been included in the **Suppl. Table 14**. It's important to note that the outcomes from Vamb were relatively unsatisfactory based on N50s, total length, #MAGs, completeness and contamination scores. Furthermore, VAMB is not designed to work at the individual sample level, which diverges from our specific application needs. We want to highlight that, to minimize any potential bias, our approach incorporated a mix of the most advanced binning tools deemed appropriate for our experiments. These tools represent the current best practices and are widely used in various pipelines, as endorsed by communities such as Nextflow and educational resources like EMBO courses.

3. Kraken2 is not a tool designed for MAG annotation, as it can only annotate single sequences, e.g. contigs. I'm curious how it was utilized to annotate MAGs.
Reply: Thank you for your question. We want to point out that Kraken2 utilizes a k-mer-based method that's effective at both the read and MAG levels. We recognize that analysis at the MAG level is particularly advantageous due to the availability of long, accurate genomic sequences, which helps in minimizing the likelihood of false positives.

4. There seems to be a lack of information on co-assembly and the quality of MAGs, such as completeness and contamination, as well as the number of low-quality, medium-quality, and high-quality bins.
Reply: We thank the reviewer for this comment and have provided the suitable stats in **Suppl. Table 13** for all MAGs.

5. I couldn't find any details on how the authors identified L. amylovorus and L. fermentum from the MAGs. Are there two strain-resolved MAGs representing these two strains? What are their completeness and contamination? It's generally quite challenging to generate strain-resolved MAGs using short-read assembly and binning.
Reply: We thank the reviewer for this comment and have now added the suitable spp. information in the last column in **Suppl. Table 13** for all MAGs: "Subsequently, we conducted taxonomy profiling of the MAGs, which revealed *L.fermentum and L.amylovorus* as abundant bacterial species in 45 and 51 samples respectively (see last column in **Suppl. Table 13**)"

References
1. Winkle, J. J. *et al*. Emergent spatiotemporal population dynamics with cell-length control of synthetic microbial consortia. PLOS Computational Biology **17**(9), e1009381. (2021).
2. Sun, J. *et al.* Quorum sensing mediates yeast cell morphology to improve settleability: Implication for wastewater treatment. Journal of Environmental Chemical Engineering **9**(5), 105817 (2021).

Reviewers' Comments:

Reviewer #2:
Remarks to the Author:
I am satisfied the authors' response

Reviewer #3:
Remarks to the Author:
All my concerns have been addressed. The paper is ready to go.

Final reviewer’s rebuttal

Reviewer #1 (Remarks to the Author):

The authors have addressed my concerns and the manuscript is ready for publication.
R: We thank the reviewer for the valuable comments.

Reviewer #2 (Remarks to the Author):

I am satisfied the authors' response.
R: We appreciate the constructive inputs the reviewer provided, and believe that they elevated the quality of the work.

Reviewer #3 (Remarks to the Author):

All my concerns have been addressed. The paper is ready to go.
R: We thank the reviewer for the suggestions, and believe they have helped to substantially improve the quality of the work.
